# A RAIR–ATC transcriptional axis and multimodal drug-response modelling reveal class-level vulnerabilities in thyroid cancer

**DOI:** 10.3389/fphar.2026.1789743

**Published:** 2026-05-18

**Authors:** Siying Wang, Dechun Zhang, Weixuan Liu, Mengqian Zhu, Rongfang Li, Zhenyu Liu, Pei Wu, Tianyu Liu

**Affiliations:** Department of Geriatrics, Changde Hospital, Xiangya School of Medicine, Central South University (The First People’s Hospital of Changde City), Changde, Hunan, China

**Keywords:** anaplastic thyroid carcinoma, drug response prediction, multimodal machine learning, radioiodine-refractory thyroid carcinoma, targeted anti-cancer drugs, transcriptional signature

## Abstract

**Introduction:**

Radioiodine-refractory papillary thyroid carcinoma (RAIR PTC) and anaplastic thyroid carcinoma (ATC) are clinically challenging thyroid cancer states, yet the molecular continuum between radioiodine refractoriness, anaplastic transformation and targeted drug sensitivity remains unclear. We hypothesised that RAIR and ATC share a common transcriptional axis that can be distilled into a transferable signature to prioritise targeted therapies in RAIR-like models.

**Methods:**

We integrated RNA-seq and microarray cohorts (TCGA-THCA, one RAIR vs. radioiodine-avid PTC cohort, and five ATC cohorts) to derive RAIR/ATC gene modules and annotate pathway activity (MSigDB Hallmark). A single-sample RAIR signature was applied to TCGA tumours and DepMap thyroid cell lines. We combined RAIR scores with PRISM drug-response AUC data (18 thyroid cell lines) to quantify differential sensitivity (ΔAUC) and trained an interpretable multimodal ridge model integrating cell and drug features to predict AUC. External transferability and pharmacology-oriented mechanisms were assessed in an independent thyroid RNA-seq cohort (GSE126698) using ssGSEA and mechanism-relevant pathways.

**Results:**

RAIR and ATC upregulated modules converged on an EMT–angiogenesis–inflammatory/interferon axis, with ATC representing a more extreme state. The RAIR signature stratified TCGA tumours with graded activation of these programs and was largely independent of BRAF and TERT promoter status, while preserving pathway associations when transferred to DepMap. In PRISM, VEGFR/KDR and RAF/BRAF inhibitor classes showed the most negative class-averaged ΔAUC values, indicating preferential activity in RAIR-high thyroid lines. The multimodal ridge model improved AUC prediction over cell-only or drug-only baselines and was concordant with class-level sensitivity patterns. In GSE126698, RAIR scores retained robust associations with hypoxia and interferon/inflammatory programs and aligned with drug-mechanism pathways including KRAS signaling up and PI3K–AKT–mTOR signaling. Initial *in vitro* validation in a single RAIR-high/ATC-like thyroid cancer model provided proof-of-concept support for these predictions, with sorafenib showing dose-dependent cytotoxicity and suppression of RAF–MAPK signalling.

**Conclusion:**

A module-based RAIR signature captures a disease-focused component of a broader RAIR–ATC axis, transfers to cell-line models and can be embedded into an interpretable multimodal framework for drug-response prediction and targeted drug-class prioritisation, prioritising VEGFR/KDR and RAF/BRAF inhibitor classes as candidates for further translational evaluation in RAIR-like thyroid models.

## 1 Introduction

Differentiated thyroid carcinoma is usually a curable disease, yet a substantial minority of patients progress to radioiodine-refractory papillary thyroid carcinoma (RAIR PTC) or anaplastic thyroid carcinoma (ATC), for whom therapeutic options are limited and prognosis is poor (Haugen et al., 2016; Karapanou et al., 2022; Volpe et al., 2024; Chen et al., 2024; Subbiah et al., 2022; Cabanillas et al., 2024). RAIR PTC is defined clinically by loss of radioiodine uptake or failure to respond to standard radioiodine therapy, and is associated with increased metastatic potential, reduced survival and a growing reliance on systemic therapies (Haugen et al., 2016; Karapanou et al., 2022; Volpe et al., 2024; Chen et al., 2024; Schubert et al., 2024). ATC, although rarer, represents the most aggressive form of thyroid cancer and is frequently preceded by a history of differentiated disease (Haugen et al., 2016; Subbiah et al., 2022; Cabanillas et al., 2024; Hebrant et al., 2012). Over the past decade, kinase inhibitors targeting VEGFR, BRAF, RET and other drivers have improved outcomes for selected patients, but responses are heterogeneous and resistance is common (Karapanou et al., 2022; Volpe et al., 2024; Chen et al., 2024; Subbiah et al., 2022; Cabanillas et al., 2024). A central challenge is that RAIR and ATC are clinically defined syndromes that likely reflect complex, multifactorial changes in differentiation, signalling and microenvironment, which are not fully captured by single-gene biomarkers.

At the molecular level, numerous studies have catalogued the mutational landscape of thyroid cancer and identified recurrent alterations in BRAF, RAS, TERT promoter and PI3K–AKT–mTOR pathway components (N. Cancer Genome Atlas Research, 2014; Colombo et al., 2020; Liu et al., 2024). More recently, transcriptomic analyses have begun to reveal how these lesions converge on broader programmes such as epithelial–mesenchymal transition, angiogenesis, immune modulation and cell-cycle dysregulation (Hebrant et al., 2012; Colombo et al., 2020; Liu et al., 2024; Yu et al., 2016; Hu et al., 2018; Wu et al., 2024). However, most studies have focused either on RAIR or on ATC in isolation, have relied on single cohorts or platforms, and have seldom interrogated their relationship to functional drug sensitivity in matched models (Hebrant et al., 2012; Colombo et al., 2020; Yu et al., 2016; Hu et al., 2018). In particular, it remains unclear whether there exists a unified “RAIR–ATC transcriptional axis” that spans differentiated and anaplastic disease, whether this axis can be distilled into a robust, transferable signature, and whether such a signature carries actionable information about targeted drug response.

Parallel advances in functional genomics and pharmacogenomics now provide an opportunity to address these questions more systematically (Tsherniak et al., 2017; Pacini et al., 2021; Corsello et al., 2020; Yang et al., 2013). Resources such as the Cancer Dependency Map (DepMap) and the PRISM drug-repurposing resource (PRISM) link large panels of cancer cell lines with genome-wide expression profiles and high-throughput drug-response screens, enabling multi-modal analyses that integrate cell state and compound properties (Tsherniak et al., 2017; Pacini et al., 2021; Corsello et al., 2020). At the same time, machine-learning methods have matured to the point where multi-modal regression models can be trained on modest-sized datasets, combining transcriptional features with drug identity or structure to predict viability readouts, as demonstrated in recent work on pharmacogenomic prediction frameworks (Sharifi-Noghabi et al., 2021; Firoozbakht et al., 2022; Chawla et al., 2022; Jiang and Li, 2024; Zhou et al., 2025). Nonetheless, the majority of existing drug-response models are pan-cancer, “black-box” and optimised for predictive performance rather than biological interpretability, and they have rarely been tailored to specific clinical entities such as RAIR thyroid cancer (Sharifi-Noghabi et al., 2021; Firoozbakht et al., 2022; Chawla et al., 2022; Jiang and Li, 2024; Zhou et al., 2025). There is a need for frameworks that start from biologically meaningful modules, yield interpretable signatures and then use machine learning as a vehicle to connect those signatures to therapeutic hypotheses.

In this study, we propose such a framework for RAIR and ATC. Using a multi-cohort design, we first perform differential expression analyses across a dedicated RAIR PTC cohort and five independent ATC cohorts to derive RAIR- and ATC-associated gene modules, and we show that these converge on a shared EMT–angiogenesis–inflammatory programme. We then collapse the RAIR module into a single-sample RAIR signature that stratifies TCGA thyroid tumours and retains its pathway-level interpretation when transferred to DepMap cell lines, where it defines RAIR-like (RAIR-high) and non-RAIR-like (RAIR-low) models. Leveraging PRISM drug-response data, we use this signature as a cell-level feature in a multi-modal ridge regression model that combines transcriptional state with drug identity to predict AUC, and we quantify RAIR-selective sensitivity using a simple differential AUC metric. Finally, we aggregate per-drug and per-class patterns and construct an integrative network that links the RAIR module, its core pathways and drug classes to RAIR-high models, prioritising VEGFR/KDR and RAF/BRAF inhibitor classes as candidates for further translational evaluation in RAIR-like thyroid models. Importantly, we complement these *in silico* analyses with *in vitro* functional validation to provide proof-of-concept validation of the predicted vulnerability of a selected RAIR-high/ATC-like model to pathway-targeted inhibition.

## 2 Methods

### 2.1 Study design and overall workflow

**FIGURE 1:**
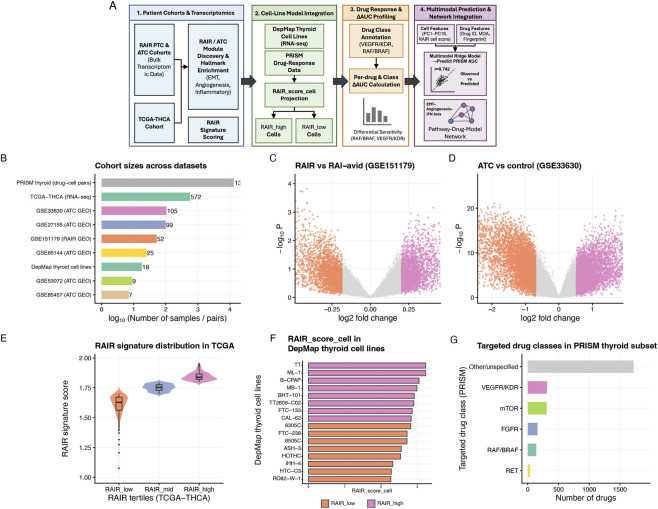
Study design and datasets used. **(A)** Schematic overview of the four-stage analytic pipeline. Bulk transcriptomic cohorts (TCGA-THCA RNA-seq and RAIR/ATC GEO datasets) are used to derive RAIR and ATC expression modules, Hallmark pathway activity, and a single-sample RAIR signature score. The signature is then projected onto DepMap thyroid cell lines, which are linked to PRISM drug-response data to map RAIR biology onto experimental models and targeted therapies, followed by ΔAUC profiling and multimodal prediction. **(B)** Cohort sizes across all datasets, shown on a log_10_ scale (raw sample or pair numbers labelled on bars). RAIR, radioiodine-refractory; ATC, anaplastic thyroid carcinoma. **(C)** Volcano plot of differential expression between RAIR and RAI-avid PTC in GSE151179 (RAIR vs. RAI-avid). Coloured points indicate genes with large positive or negative log_2_ fold change. **(D)** Volcano plot of differential expression between ATC and control in GSE33630 (ATC vs. control). **(E)** Distribution of RAIR signature scores across tertiles in TCGA-THCA (RAIR_low, RAIR_mid, RAIR_high). **(F)** RAIR_score_cell in DepMap thyroid cell lines, sorted from high to low. Bars are coloured by RAIR-like group (RAIR_high vs. RAIR_low). **(G)** Number of targeted compounds per drug class in the PRISM thyroid subset, with bars coloured by annotated drug class (RAF/BRAF, FGFR, VEGFR/KDR, mTOR, RET, Other/unspecified).

**FIGURE 2:**
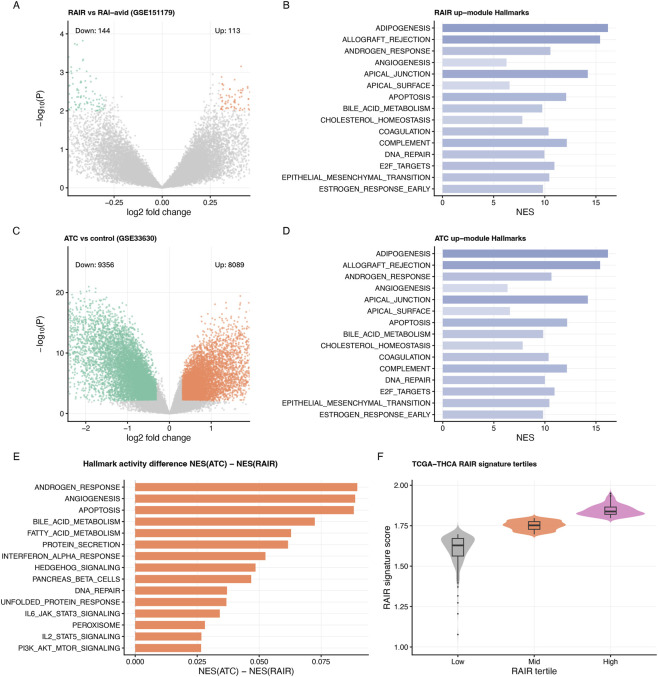
RAIR and ATC modules and their Hallmark pathway activity. **(A)** Volcano plot for the RAIR GEO cohort GSE151179 (RAIR vs. RAI-avid). Upregulated and downregulated genes are highlighted in colour. **(B)** Hallmark enrichment of the RAIR up-module (RAIR_up), showing normalized enrichment scores (NES) for the top 15 pathways (fgsea). **(C)** Volcano plot for the ATC cohort GSE33630 (ATC vs. control). **(D)** Hallmark enrichment of the ATC up-module (ATC_up). **(E)** Difference in Hallmark activity between ATC and RAIR modules, NES(ATC) − NES(RAIR), for the most affected pathways, highlighting angiogenesis, apoptosis, inflammatory and interferon responses, and cell-cycle–related programs. **(F)** RAIR signature scores across tertiles in TCGA-THCA, confirming a monotonic increase from RAIR_low to RAIR_high and establishing the module-derived signature in patient tumours before its transfer to DepMap cell lines for subsequent machine-learning analyses.

**FIGURE 3:**
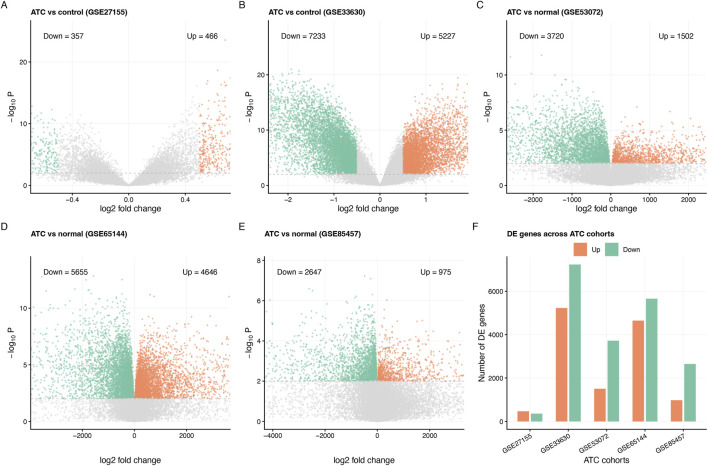
Consistency of ATC transcriptional changes across multiple cohorts. **(A–E)** Volcano plots of differential expression for five independent ATC GEO cohorts (GSE27155, GSE33630, GSE53072, GSE65144 and GSE85457), comparing ATC with PTC or adjacent normal tissue as indicated. Coloured points mark strongly upregulated and downregulated genes; grey points indicate genes with smaller or non-significant changes. **(F)** Number of upregulated and downregulated genes across the five ATC cohorts, illustrating broadly consistent transcriptional shifts despite platform and cohort differences. Together, these data support the robustness of the ATC expression module.

**FIGURE 4:**
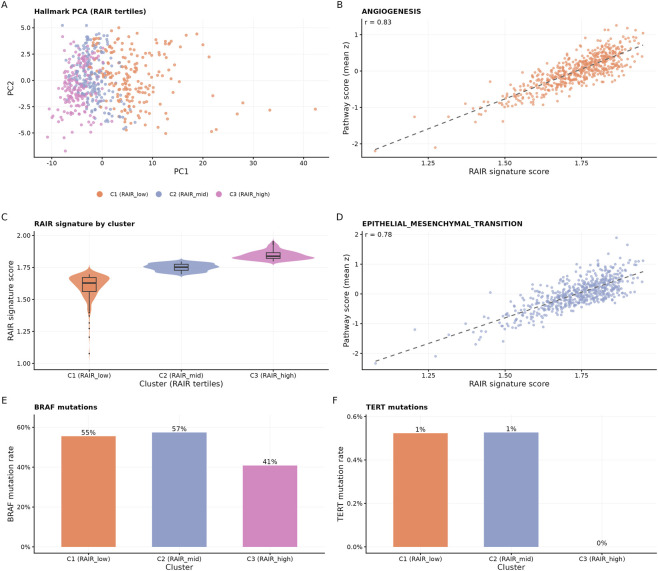
TCGA RAIR signature tertiles and their Hallmark and mutational features. **(A)** Principal component analysis of Hallmark pathway scores in TCGA-THCA. Each point is a tumour sample, coloured by RAIR tertile cluster (C1, RAIR_low; C2, RAIR_mid; C3, RAIR_high), with the corresponding colour key shown in the panel. **(B)** Scatter plot of RAIR signature score versus Hallmark ANGIOGENESIS score, with Pearson correlation coefficient (r) indicated. **(C)** Violin and box plots of RAIR signature scores across the three RAIR tertile clusters, showing a clear separation of RAIR_low, RAIR_mid and RAIR_high groups. **(D)** Scatter plot of RAIR signature score versus Hallmark EPITHELIAL_MESENCHYMAL_TRANSITION (EMT) score, linking RAIR-high tumours to EMT-like programs. **(E)** BRAF mutation rates within each RAIR tertile cluster, expressed as percentages. **(F)** TERT promoter mutation rates within each cluster. Mutation frequencies are broadly comparable, suggesting that the RAIR signature primarily captures pathway-level differences beyond individual driver mutations.

This study was designed to connect a transcriptional axis of radioiodine-refractory disease with anaplastic transformation and targeted drug sensitivity across bulk tumours and cell-line models. We first used bulk expression cohorts—TCGA-THCA, a RAIR versus radioiodine-avid PTC cohort, and five independent ATC cohorts—to perform differential expression and derive RAIR- and ATC-associated gene modules (Figures 1A,B; Table 1). These modules were then mapped onto MSigDB Hallmark pathways to identify a shared EMT/angiogenesis/inflammatory programme, and collapsed into a single-sample RAIR signature score that stratifies TCGA tumours along this axis (Figures 1C–E; Figures 2–4).

**TABLE 1:** Summary of datasets used in this study.

Dataset	Datatype	Platform	N	Histology	Role
TCGA-THCA	RNA-seq + multi-omics	TCGA	572	PTC/FTC/ATC	Define and validate RAIR signature
GSE151179	GEO expression	GEO	52	PTC	Define RAIR modules
GSE27155	GEO expression	GEO	99	ATC/PTC/Normal	ATC differential analysis
GSE33630	GEO expression	GEO	105	ATC/PTC/Normal	ATC differential analysis
GSE53072	GEO expression	GEO	9	ATC/PTC/Normal	ATC differential analysis
GSE65144	GEO expression	GEO	25	ATC/PTC/Normal	ATC differential analysis
GSE85457	GEO expression	GEO	7	ATC/PTC/Normal	ATC differential analysis
GSE126698	RNA-seq	GEO	28	Normal/PTC/FTC/ATC	External validation (pathway- and mechanism-level)
DepMap thyroid cell lines	RNA-seq	DepMap/CCLE	18	Thyroid cancer cell lines	Derive RAIR score and pathway mapping
PRISM thyroid subset	Drug response (AUC)	PRISM	13,070	Thyroid cancer cell lines	Compare drug sensitivity in RAIR-high vs. RAIR-low cells

DepMap, Cancer Dependency Map; CCLE, cancer cell line encyclopedia; RAIR, radioiodine-refractory; ATC, anaplastic thyroid carcinoma; PTC, papillary thyroid carcinoma; FTC, follicular thyroid carcinoma.

Overview of all datasets analysed. For each dataset, the table lists the data type (bulk RNA-seq or GEO expression microarray for tumours; PRISM drug-response AUC for cell lines), platform/source, number of samples or cell–drug pairs (N), major histology context, and the primary analytical role in the study. RAIR, radioiodine-refractory; ATC, anaplastic thyroid carcinoma; PTC, papillary thyroid carcinoma; FTC, follicular thyroid carcinoma.

**FIGURE 5:**
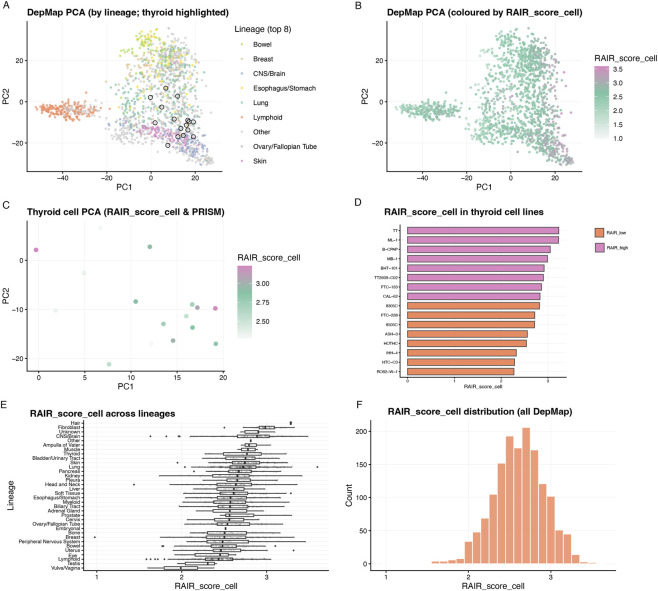
RAIR_score_cell landscape across DepMap and thyroid cell-line models. **(A)** DepMap cell-line PCA (PC1/PC2) coloured by tissue lineage. Thyroid cell lines are highlighted by open black circles, showing their position within the global landscape. **(B)** Same PCA as in **(A)** coloured by RAIR_score_cell, with the colour scale shown in the panel. **(C)** PCA restricted to PRISM-profiled thyroid cell lines, coloured by RAIR_score_cell. **(D)** RAIR_score_cell values for individual DepMap thyroid cell lines, sorted from high to low. Bars are coloured by RAIR-like group. **(E)** Distribution of RAIR_score_cell across lineages in DepMap, illustrating that RAIR-like transcriptional states occur across multiple cancer types. **(F)** Histogram of RAIR_score_cell across all DepMap cell lines.

**FIGURE 6:**
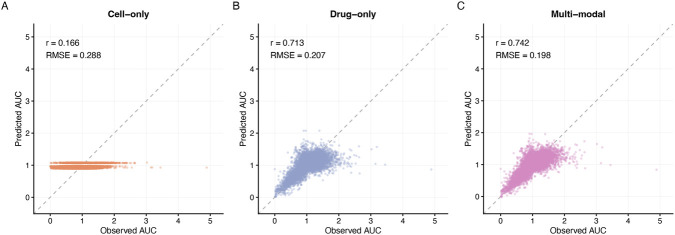
Performance of machine-learning models for PRISM AUC prediction in thyroid cell lines **(A–C)** Scatter plots of observed versus predicted AUC for the cell-only model **(A)** drug-only model **(B)** and multi-modal model combining cell and drug features **(C)**. Diagonal dashed lines indicate perfect prediction. Pearson correlation coefficients and root-mean-square error (RMSE) are reported in each panel. Using sixteen expression principal components alone yields modest performance (r = 0.166; RMSE = 0.288), whereas a drug-only model based on one-hot encoded compound identity explains most of the variance (r = 0.713; RMSE = 0.207). The multi-modal model provides a small but consistent improvement (r = 0.742; RMSE = 0.198). All models were implemented using scikit-learn with standardised features and evaluated by 5-fold cross-validation on 13,070 thyroid cell–drug pairs. Lower PRISM AUC indicates stronger growth inhibition.

**FIGURE 7:**
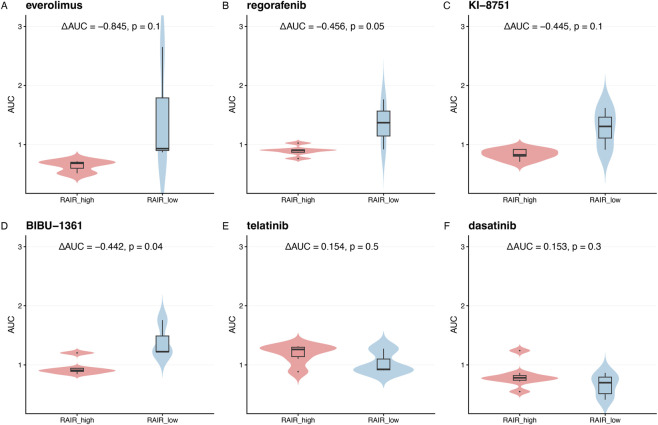
Selected targeted agents with differential activity in RAIR-like versus non-RAIR-like thyroid cell lines. Violin and box plots show AUC distributions in RAIR_high versus RAIR_low DepMap thyroid cell lines for six representative targeted agents. **(A)** Everolimus (mTOR inhibitor). **(B)** Regorafenib (multi-kinase inhibitor). **(C)** KI-8751 (VEGFR inhibitor). **(D)** BIBU-1361 (VEGFR/PDGFR inhibitor). **(E)** Telatinib (VEGFR inhibitor). **(F)** Dasatinib (SRC/ABL inhibitor). For each drug, the annotation reports ΔAUC (RAIR_high − RAIR_low) and the p-value from a Wilcoxon test; negative ΔAUC indicates greater sensitivity (lower AUC) in RAIR_high cells. These per-drug ΔAUC values are also used as ground-truth labels for training and evaluating the multi-modal machine-learning models in Figure 6.

**FIGURE 8:**
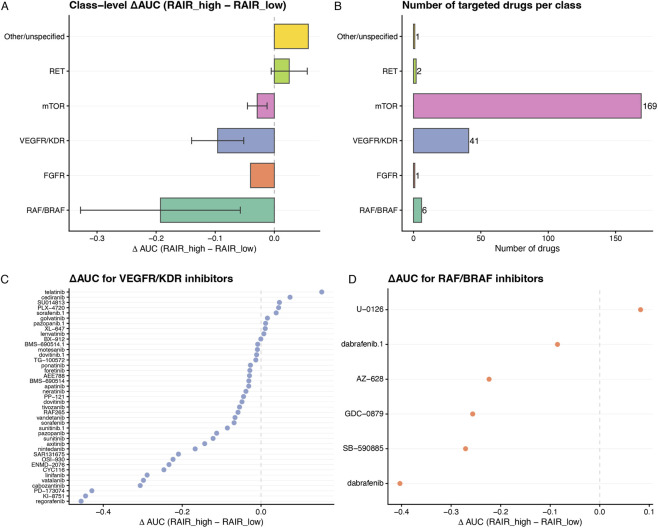
Drug-class level RAIR sensitivity and per-drug ΔAUC. **(A)** Class-level ΔAUC (RAIR_high − RAIR_low) for targeted drug classes (RAF/BRAF, FGFR, VEGFR/KDR, mTOR, RET, Other/unspecified). Bars show mean ΔAUC with 95% confidence intervals; negative values indicate overall greater sensitivity in RAIR_high cells. **(B)** Number of targeted drugs per class in the PRISM thyroid subset. **(C)** Per-drug ΔAUC for VEGFR/KDR inhibitors, ordered from most RAIR_high-sensitive (most negative) to least. **(D)** Per-drug ΔAUC for RAF/BRAF inhibitors, highlighting several agents with preferential activity in RAIR_high cell lines. These class- and drug-level ΔAUC landscapes were used both to nominate VEGFR/KDR and RAF/BRAF inhibitors as promising candidates and as supervision targets for the multi-modal machine-learning models (Figure 6) and the integrated network in Figure 9.

**FIGURE 9:**
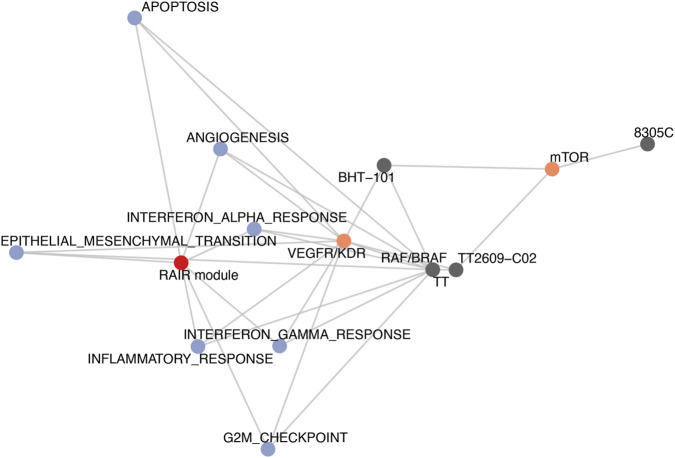
Integrated RAIR module–Hallmark–drug class–model network. Compact network representation integrating RAIR and ATC modules, Hallmark enrichments, drug-class effects and RAIR-like thyroid models. Nodes represent the RAIR module (red), Hallmark pathways (blue), targeted drug classes (orange) and RAIR_high thyroid cell lines with PRISM coverage (grey). Edges from drug classes to RAIR_high cell lines are weighted by relative sensitivity (based on class-level ΔAUC and multi-modal model predictions), highlighting a RAIR module → EMT/interferon/inflammatory/angiogenesis core → VEGFR/KDR and RAF/BRAF inhibitor axis in RAIR_high experimental models.

In the second stage, we transferred the RAIR signature from patients to models. We computed a RAIR score for each DepMap cell line, defined RAIR-like thyroid cell lines (RAIR-high versus RAIR-low), and integrated these with PRISM high-throughput drug screening data to obtain AUC and ΔAUC readouts for ∼1,500 compounds in 18 thyroid cell lines (Figures 1F,G, 5; Supplementary Figure S1; Supplementary Table S1). Finally, we built regularised multi-modal machine-learning models that combine cell-level transcriptional features (expression PCs and RAIR cell score) with drug identity to predict PRISM AUC (Figure 6). Per-drug and per-class ΔAUC patterns, together with model predictions, were aggregated into class-level summaries and an integrative network linking the RAIR module, Hallmark pathways, drug classes and RAIR-high cell lines (Figures 7–9; Supplementary Table S2).

### 2.2 Bulk RNA-seq and microarray datasets

RNA-seq data for primary thyroid tumours were obtained from The Cancer Genome Atlas thyroid carcinoma project (TCGA-THCA) via the Genomic Data Commons/UCSC Xena public releases (N. Cancer Genome Atlas Research, 2014). We used the gene-level STAR count and TPM matrices together with associated clinical and somatic mutation annotations. Only primary tumour samples with matched RNA-seq and clinical information were retained. Lowly expressed genes were first filtered from the raw count matrix using the filterByExpr function in edgeR, which adaptively determines an expression cutoff based on library sizes and the group structure so that only genes with sufficient counts in an adequate number of samples are kept for downstream analysis. For expression-level analyses (e.g., signature scoring and correlation with Hallmark pathways), TPM values were then transformed as log2 (TPM +1). BRAF and TERT promoter mutation status was derived from the somatic mutation calls by flagging non-synonymous BRAF substitutions and canonical TERT promoter hotspot variants (Barrett et al., 2013).

For the radioiodine-refractory discovery cohort, we downloaded the GSE151179 series from the NCBI Gene Expression Omnibus (GEO), which profiled papillary thyroid carcinomas classified as RAIR or radioiodine-avid according to the original study (Colombo et al., 2020; Barrett et al., 2013). Processed expression matrices provided by the authors were used where available; otherwise, raw counts were normalised using standard RNA-seq workflows (library-size normalisation and log2 transformation). Samples were included if they had unambiguous RAIR or radioiodine-avid labels and passed basic quality control (no extreme library sizes, no obvious outliers in principal component analysis).

To capture the anaplastic phenotype, we used five ATC-enriched GEO cohorts: GSE27155, GSE33630, GSE53072, GSE65144 and GSE85457 (Hebrant et al., 2012; Yu et al., 2016; Hu et al., 2018; Barrett et al., 2013). For microarray-based studies, raw CEL files or series matrices were imported into R and processed with robust multi-array average normalisation, followed by log2 transformation. Probe sets were mapped to gene symbols using the corresponding GPL platform annotations; when multiple probes mapped to the same gene, their expression was summarised by median or mean intensity. Cohorts were restricted to samples annotated as ATC, differentiated PTC or non-malignant thyroid tissue, and were used in pairwise contrasts of ATC versus PTC or ATC versus normal thyroid for differential expression and module derivation. For independent external validation, we additionally downloaded GSE126698 from GEO, an RNA-seq cohort comprising normal thyroid tissue, papillary thyroid carcinoma, follicular thyroid carcinoma and anaplastic thyroid carcinoma samples. Expression values (FPM) were log1p-transformed prior to signature and pathway scoring, and gene identifiers were mapped to HGNC symbols as provided.

### 2.3 Differential expression and module definition

For all bulk cohorts, differential expression was performed in R using limma (Ritchie et al., 2015). For RNA-seq data (TCGA and RNA-seq GEO cohorts), voom was applied to log-transformed counts to estimate the mean–variance relationship and generate precision weights; for microarray cohorts, robust multi-array average normalisation was used directly (Law et al., 2014). In each cohort, a linear model was fitted with a group factor (for example, RAIR versus radioiodine-avid PTC, or ATC versus PTC/normal), and empirical Bayes moderation was applied to obtain moderated t statistics, P values and log_2_ fold changes. To define differentially expressed genes, we used a primary threshold of P < 1 × 10^−2^ and absolute log_2_ fold change >0.5. These thresholds were chosen to yield stable gene sets across RAIR and ATC cohorts while controlling the number of spurious hits; we additionally report Benjamini–Hochberg adjusted P values for reference.

RAIR modules were derived from the RAIR PTC discovery cohort. Genes significantly upregulated in RAIR versus radioiodine-avid tumours were collected into a RAIR-up module, and those significantly downregulated into a RAIR-down module, after mapping probe identifiers to unique gene symbols and removing genes without unambiguous annotation. For ATC modules, we first computed differential expression in each ATC cohort separately using the same thresholds. We then mapped all results to gene symbols and defined ATC-up and ATC-down modules as the union of genes significantly up- or downregulated in at least one ATC study. To reduce platform-specific noise, genes that appeared only once with marginal effect sizes were down-weighted in exploratory analyses, but the primary module definitions are based on the union to maximise sensitivity to recurrent ATC-associated changes. These four modules (RAIR-up, RAIR-down, ATC-up and ATC-down) were used for subsequent enrichment, signature and transfer analyses.

### 2.4 Hallmark pathway scoring and correlation analyses

To interpret RAIR and ATC modules at the pathway level, we used the Molecular Signatures Database (MSigDB) Hallmark gene sets (human, symbol version) (Liberzon et al., 2015). For module-level enrichment, genes were ranked by the moderated statistic or log_2_ fold change within each contrast, and fast gene set enrichment analysis was performed with fgsea (Korotkevich et al., 2016). Normalised enrichment scores, nominal P values and Benjamini–Hochberg adjusted P values were reported for each Hallmark. Enrichment analyses were carried out separately for the RAIR-up and ATC-up modules and for each bulk cohort, but downstream interpretation focused on the consolidated RAIR- and ATC-associated rankings.

For sample-level pathway activity, we computed Hallmark scores in both TCGA and DepMap. Expression matrices were first transformed to gene-wise z scores across samples. For each Hallmark set, a pathway score was then calculated as the mean z score of its member genes in each sample (equivalent to a simple, transparent single-sample gene set enrichment measure). This yielded a sample-by-Hallmark score matrix for TCGA tumours and for cell lines. We then correlated the RAIR signature score or RAIR cell score (see below) with each Hallmark score using Pearson correlation, obtaining r values for the patient cohort (r_TCGA) and for DepMap (r_DepMap). Differences in correlation between the two contexts were summarised as Δr = r_DepMap − r_TCGA for descriptive purposes. These statistics were used to rank pathways by strength and consistency of association with the RAIR axis. In the external RNA-seq cohort (GSE126698), pathway activities were computed using single-sample GSEA (ssGSEA; GSVA) on log1p-transformed expression values to improve robustness across platforms.

To further assess whether the RAIR signature might be confounded by bulk tumour composition, we performed supplementary tumour-purity and tumour microenvironment-oriented analyses. In the discovery cohort GSE151179, StromalScore, ImmuneScore, ESTIMATEScore and TumorPurity were calculated using the ESTIMATE framework and compared between RAI-avid and RAIR tumours. In TCGA-THCA, the RAIR signature score was additionally correlated with marker-based scores for endothelial cells, macrophages, B cells, T cells and fibroblasts, together with ESTIMATE-derived ImmuneScore, StromalScore, ESTIMATEScore and TumorPurity. Marker-based scores were calculated as the mean gene-wise z score of predefined marker sets. Correlations were evaluated using Pearson correlation, and multiple testing was controlled by the Benjamini–Hochberg procedure. These supplementary analyses are summarised in Supplementary Figure S8 and Supplementary Table S3.

### 2.5 RAIR signature and RAIR cell score

In the TCGA-THCA cohort, a RAIR signature score was constructed for each tumour using the RAIR-up module. After log_2_ (TPM +1) transformation and gene-wise z standardisation, the RAIR signature for a given sample was defined as the arithmetic mean of z scores across RAIR-up genes present in TCGA. This simple module-based aggregate emphasises coherent upregulation of RAIR-associated genes and down-weights idiosyncratic changes. For most analyses, we treated the RAIR signature as a continuous variable. For stratification, tumours were grouped into tertiles according to their RAIR score to yield RAIR-low, RAIR-mid and RAIR-high strata of approximately equal size. These strata were then used for comparisons of pathway activity and mutation frequencies.

To transfer the RAIR axis to cell lines, we computed an analogous RAIR cell score in the DepMap expression matrix. The same RAIR-up gene list was mapped to gene symbols in DepMap RNA-seq data. Gene-wise z scores were computed across all cell lines, and the RAIR cell score (RAIR_score_cell in code) was defined as the mean z score of RAIR-up genes for each line. This produced a continuous score per cell line that can be interpreted as the degree to which the RAIR module is engaged. For lineage-level summaries, scores were compared across DepMap tissue categories, and for thyroid analyses, the 18 thyroid cell lines were dichotomised at the median RAIR cell score into RAIR-low and RAIR-high groups. These groups served as the basis for ΔAUC calculations and for selecting RAIR-like models for network analysis. The same RAIR-up gene list was applied unchanged to the external cohort (GSE126698) to compute RAIR signature scores.

### 2.6 PRISM drug-response data and ΔAUC definition

High-throughput drug response data were obtained from the PRISM secondary screen release (Corsello et al., 2020). We restricted the analysis to cell–drug pairs involving the 18 DepMap thyroid cell lines and compounds with valid AUC estimates. PRISM AUC represents the area under the dose–response viability curve, with lower values indicating stronger growth inhibition. Pairs with missing or extreme quality control flags were removed. For descriptive summaries, we tabulated, for each cell line, the number of compounds tested and the distribution of AUC values, and for each compound, the number of thyroid lines profiled and its mean AUC across these lines.

To distinguish mechanism-based agents from non-specific cytotoxics, we annotated compounds as “targeted-like” or “other/unspecified” using PRISM-provided mechanism of action and target fields. Drugs whose target or mechanism text contained canonical kinase or receptor names (for example, EGFR, VEGFR, FGFR, RAF, BRAF, RET, MET, PI3K, AKT, mTOR, SRC) were labelled targeted-like; all others were grouped as other/unspecified. This coarse categorisation was used solely for global comparisons of AUC distributions and for defining drug classes in subsequent class-level analyses.

To quantify RAIR-selective drug response, we defined for each compound a differential AUC, ΔAUC, between RAIR-high and RAIR-low thyroid cell lines. Specifically, ΔAUC was computed as the mean AUC across RAIR-high lines minus the mean across RAIR-low lines. Negative ΔAUC indicates that, on average, the drug is more potent in RAIR-high models (lower viability AUC), whereas positive values indicate relative resistance. For each drug, we also performed a two-sided Wilcoxon rank-sum test comparing AUC values between the 2 cell groups. These per-drug ΔAUC and P values were used to identify representative compounds, to summarise class-level sensitivity by averaging ΔAUC within mechanism classes, and to provide ground-truth labels and evaluation metrics for the multi-modal machine-learning models.

### 2.7 Multi-modal machine-learning models for PRISM AUC

To model drug response as a function of both cell state and drug identity, we built regularised linear regression models using features from DepMap expression profiles and PRISM compound annotations, following general principles from recent pharmacogenomic modelling studies (Sharifi-Noghabi et al., 2021; Firoozbakht et al., 2022; Chawla et al., 2022; Jiang and Li, 2024; Zhou et al., 2025). Let $y\in R^{N}$ denote the vector of PRISM AUC values for N thyroid cell–drug pairs, and $X\in R^{N\times P}$ the corresponding feature matrix. For each pair, the feature vector was constructed by concatenating cell-level features and drug-level features.

Cell-level features were derived from the DepMap expression matrix by principal component analysis (Tsherniak et al., 2017; Pacini et al., 2021). Gene expression values (log-transformed and centred) across all cell lines were projected onto the first 16 principal components, which captured the majority of variance in the expression space. For thyroid-specific analyses, we also included the RAIR cell score as an additional feature. Drug-level features were encoded using a one-hot representation of the compound identifier, with one binary indicator per unique drug. In supplementary analyses, one-hot drug features were optionally replaced by 1,024-bit Morgan fingerprints computed from compound SMILES (Landrum, 2013), but these fingerprint-based models were used only as sensitivity checks. Before model fitting, each feature column $X_{.j}$ was standardised to zero mean and unit variance. The feature standardization, ridge-regression objective, RMSE, and Pearson correlation coefficient are defined in Equations 1–4.

$$X_{ij}=\frac{X_{ij}-\mu_{j}}{\sigma_{j}}$$ (1)

where $\mu_{j}$ and $\sigma_{j}$ are the mean and standard deviation of feature j across all training samples. Models were implemented as ridge regressions, i.e., L2-regularised linear models. For a given feature set, model parameters β were estimated by minimising:

$$\min_{\beta }{\left\|y-X\beta \right\|}_{2}^{2}+{\lambda \left\|\beta \right\|}_{2}^{2}$$ (2)

where λ > 0 is a regularisation strength. In practice, we used the default ridge implementation in scikit-learn with a fixed λ (Pedregosa et al., 2011), and considered three configurations: (i) a cell-only model using only cell-level principal components (PCs; and the RAIR cell score); (ii) a drug-only model using only one-hot drug identity features; and (iii) a multi-modal model combining both cell- and drug-level features.

For each configuration, we performed five-fold cross-validation on the thyroid PRISM dataset. On each fold, the model was trained on 80% of the cell–drug pairs and evaluated on the held-out 20%; predictions from all folds were concatenated to obtain cross-validated predictions ${\hat{y}}_{i}^{\left(cv\right)}$ . Predictive performance was quantified by the root-mean-square error (RMSE):

$$RMSE=\sqrt{\frac{1}{N}\sum_{i=1}^{N}{\left(y_{i}-{\hat{y}}_{i}^{\left(cv\right)}\right)}^{2}}$$ (3)

and the Pearson correlation coefficient between observed and predicted AUC:

$$r=\frac{\sum_{i}^{N}\left(y_{i}-\bar{y}\right)\left({\hat{y}}_{i}^{\left(cv\right)}-\bar{{\hat{y}}_{i}^{\left(cv\right)}}\right)}{\sqrt{\sum_{i=1}^{N}{\left(y_{i}-\bar{y}\right)}^{2}}\sqrt{\sum_{i=1}^{N}{\left({\hat{y}}_{i}^{\left(cv\right)}-\bar{{\hat{y}}_{i}^{\left(cv\right)}}\right)}^{2}}}$$ (4)

These same cross-validated predictions were also used to compare model-derived sensitivity patterns with experimentally observed per-drug and per-class ΔAUC in downstream analyses.

Because the DepMap–PRISM framework is based on monoculture cell-line screens, the multimodal model was designed to capture tumour-cell-associated drug-response variation under standardised *in vitro* conditions. It therefore does not explicitly incorporate patient-level tumour microenvironment variables, such as immune infiltration, stromal Supplementary Figure S9 context or local hypoxia severity, which may further modulate inhibitor efficacy *in vivo*. We therefore interpret the current framework as a prioritisation model for intrinsic vulnerabilities and contextualise its predictions using external pathway- and microenvironment-oriented analyses (Supplementary Figures S5, S6 and S8 and Supplementary Table S3).

To assess the incremental value and disease specificity of the study-derived RAIR signature, we benchmarked it against five related transcriptional signatures: EMT, Hypoxia, Angiogenesis, Inflammatory Response and IFN-gamma Response. In the public-cohort benchmark, each signature was scored in GSE151179 to evaluate discrimination between RAIR and RAI-avid tumours using signed AUC, and then transferred to four independent ATC public cohorts to assess separation magnitude and direction consistency across ATC contrasts. In the DepMap/PRISM thyroid subset, we projected each signature to thyroid cell lines, compared class-averaged ΔAUC values for RAF/BRAF and VEGFR/KDR inhibitor classes between signature-defined high and low models, and evaluated predictive gain by adding each signature separately to the drug-only PRISM baseline model and calculating the change in cross-validated *R* ^2^. These benchmark analyses are summarised in Supplementary Figure S9 and Supplementary Table S4.

### 2.8 External validation and pharmacology-oriented mechanism analyses

To provide independent validation, we applied the RAIR signature to GSE126698 and examined (i) associations between RAIR scores and selected Hallmark ssGSEA programs (hypoxia, interferon/inflammatory responses, EMT and related pathways) and (ii) pharmacology-oriented mechanisms relevant to the prioritised drug classes. Specifically, we tested correlations between RAIR scores and target-axis gene expression (VEGF/VEGFR and RAF–MEK–ERK axes) and between RAIR scores and drug-mechanism Hallmark pathways (angiogenesis, KRAS signaling up and PI3K–AKT–mTOR signaling), using Pearson correlation with BH-FDR correction.

### 2.9 *In vitro* validation experiments

CAL-62 thyroid cancer cells were used as a RAIR-high/ATC-like model based on DepMap transcriptomic analyses. For viability assays, cells were seeded in 96-well plates (3,000 cells per well) and treated with a log-spaced concentration series of sorafenib (10-point dilution spanning 0.03–30 μM) with vehicle (DMSO) controls. After 72 h, cell viability was measured using an MTT assay and normalised to vehicle-treated controls. Dose–response curves were fitted with a four-parameter logistic model to estimate IC50.

For pathway inhibition experiments, CAL-62 cells were treated with sorafenib (0, 1, 5 and 12 μM) for 6 h. Whole-cell lysates were prepared and analysed by immunoblotting for EGFR and phosphorylated EGFR (p-EGFR), ERK and phosphorylated ERK (p-ERK), and BRAF and phosphorylated BRAF (p-BRAF), with β-actin or GAPDH used as loading controls. Band intensities were quantified using ImageJ. For phosphorylated proteins, phosphorylation ratios were calculated relative to the corresponding total protein levels (p-EGFR/EGFR, p-ERK/ERK and p-BRAF/BRAF), and each ratio was then normalised to the untreated control.

### 2.10 Statistical analysis and software

Unless otherwise specified, all statistical analyses and visualisations were performed in R (version 4.4.2) (Ritchie et al., 2015; Law et al., 2014; Liberzon et al., 2015; Korotkevich et al., 2016). Differential expression analyses used the limma package, with voom applied for RNA-seq count data and robust multi-array average for microarrays. Module-level and Hallmark enrichment analyses used fgsea, and sample-level Hallmark scores were computed either by mean z-score aggregation or single-sample gene set scoring. Group comparisons of continuous variables (e.g., AUC between RAIR-high and RAIR-low cell lines) primarily used two-sided Wilcoxon rank-sum tests. Correlations between RAIR scores and pathway activities were quantified using Pearson’s correlation coefficient; where appropriate, both the correlation coefficient and corresponding P value were reported. Multiple testing was controlled by the Benjamini–Hochberg procedure, with a false discovery rate threshold of 0.05 unless otherwise noted.

Machine-learning models were implemented in Python using scikit-learn, with additional scientific computing support from NumPy and pandas (Landrum, 2013; Pedregosa et al., 2011). Compound fingerprints in supplementary analyses were generated using RDKit. Network visualisations were created in R using tidygraph and ggraph, and other plots were produced with ggplot2 and patchwork. All analyses were scripted to ensure reproducibility, and key intermediate objects (e.g., module gene lists, RAIR signature scores, RAIR cell scores and PRISM feature matrices) were stored as RDS or CSV files for reuse across analyses.

## 3 Results

### 3.1 Multi-cohort bulk analysis of RAIR and ATC

To delineate a transcriptional axis linking radioiodine-refractory PTC and anaplastic transformation, we first assembled a panel of bulk tumour cohorts across the spectrum of thyroid cancer (Hebrant et al., 2012; N. Cancer Genome Atlas Research, 2014; Colombo et al., 2020; Yu et al., 2016; Hu et al., 2018). As summarised in the workflow and dataset overview (Figures 1A,B; Table 1), the discovery set comprised 572 TCGA-THCA tumours, a dedicated RAIR PTC cohort (GSE151179, 52 cases) and five ATC-enriched cohorts (GSE27155, GSE33630, GSE53072, GSE65144 and GSE85457; 99, 105, 9, 25 and 7 cases, respectively). These datasets provide complementary contrasts—RAIR versus radioiodine-avid PTC, and ATC versus differentiated or normal thyroid—allowing us to search for shared patterns of transcriptional change.

In the RAIR PTC cohort, differential expression analysis revealed broad transcriptional rewiring in RAIR tumours compared with radioiodine-avid controls. The volcano plot (Figure 1C, re-scaled in Figure 2A) shows hundreds of genes significantly altered at P < 10^–2^ with |log_2_ fold change| > 0.5. We grouped significantly upregulated genes into a RAIR-up module and downregulated genes into a RAIR-down module, forming a compact RAIR-associated gene set that we later collapse into a continuous RAIR score in TCGA and cell lines.

Parallel analyses in the five ATC cohorts yielded even more dramatic perturbations. Volcano plots in Figures 1D, 3A–E highlight thousands of genes up- or downregulated at the same thresholds, despite differences in comparator tissues (PTC vs. normal). Across studies, ATC profiles consistently show loss of thyroid-lineage markers and induction of proliferative and stress-response programmes. Counting differentially expressed genes confirms that each cohort contributes hundreds to thousands of up- and downregulated genes (Figure 3F). From these, we defined ATC-up and ATC-down modules as the union of recurrently altered genes. Together, these RAIR and ATC modules provide a modular summary of disease-associated transcriptional states for downstream pathway analysis, signature construction and functional transfer.

### 3.2 Hallmark analysis defines a common EMT–angiogenesis–inflammatory programme

We next asked whether the RAIR and ATC modules converge on shared biological pathways. Projection of the RAIR-up module onto 50 MSigDB Hallmark gene sets revealed strong enrichment for epithelial–mesenchymal transition (EMT), angiogenesis, inflammatory and interferon responses, apoptosis, complement/coagulation and G2M checkpoint control (Figure 2B). Normalised enrichment scores were high, indicating that a substantial fraction of RAIR-up genes participate in these coordinated programmes rather than being functionally scattered.

Applying the same analysis to the ATC-up module yielded a highly similar pattern (Figure 2D). Epithelial–mesenchymal transition, angiogenesis, inflammatory response, interferon-alpha/gamma responses, apoptosis, complement and G2M checkpoint again ranked among the top Hallmarks, but with slightly higher enrichment scores and broader gene coverage in ATC—consistent with ATC representing a more extreme state along the same axis. Direct comparison of enrichment scores (NES(ATC) − NES(RAIR); Figure 2E) showed that androgen response, angiogenesis, apoptosis, fatty acid metabolism, unfolded protein response and several interferon-related pathways are modestly more activated in ATC than in RAIR, whereas PI3K/AKT/mTOR signalling differs little.

To test whether this programme also explains inter-patient variability and is preserved in models, we correlated a sample-level RAIR score with Hallmark activities in TCGA and DepMap. The top pathways in TCGA (UV response down, apical surface, hypoxia, apoptosis, angiogenesis, G2M checkpoint, EMT, inflammatory and interferon responses) all showed strong positive correlations with the RAIR score (typically r_TCGA 0.7–0.9; Table 2). The same pathways also correlated with RAIR score in DepMap thyroid cell lines with r_DepMap generally 0.6–0.8 (Table 2; Supplementary Figure S3A). The heatmap and Δr bar plot in Supplementary Figure S3A–B illustrate that EMT/angiogenesis/inflammatory Hallmarks have consistent direction and only modest correlation differences between patients and cell lines. Overall, these results define a robust RAIR–ATC transcriptional axis dominated by EMT, neovascularisation, inflammatory and interferon signalling and G2M activity, which we use as the biological backbone for stratification and modelling.

**TABLE 2:** Top Hallmark pathways associated with the RAIR signature in patients and cell lines.

Hallmark	rTCGA	rDepMap	deltaR
UV_RESPONSE_DN	0.92	0.78	−0.14
APICAL_SURFACE	0.91	0.73	−0.19
HYPOXIA	0.91	0.78	−0.13
APOPTOSIS	0.88	0.87	−0.01
ANGIOGENESIS	0.83	0.6	−0.23
G2M_CHECKPOINT	0.81	0.34	−0.47
EPITHELIAL_MESENCHYMAL_TRANSITION	0.78	0.6	−0.18
INFLAMMATORY_RESPONSE	0.76	0.76	−0.01
INTERFERON_GAMMA_RESPONSE	0.71	0.71	0
INTERFERON_ALPHA_RESPONSE	0.71	0.69	−0.02

Top Hallmark pathways ranked by the absolute Pearson correlation between the RAIR, signature score and pathway activity in TCGA-THCA (rTCGA). For each pathway, the table reports the correlation in TCGA, tumours (rTCGA), the corresponding correlation in DepMap cell lines (rDepMap), and the difference in correlation (deltaR = rDepMap − rTCGA). Pathways include EMT, angiogenesis, apoptosis, inflammatory and interferon responses, G2M checkpoint and others, illustrating robust and largely concordant RAIR, biology across patient samples and cell-line models.

### 3.3 External validation and pharmacology-oriented mechanism support in GSE126698

To assess transferability beyond the discovery datasets, we applied the same 300-gene RAIR-up module to an independent thyroid RNA-seq cohort (GSE126698) comprising normal thyroid tissue, papillary, follicular and anaplastic thyroid carcinomas. RAIR signature scores showed heterogeneous distributions across histological categories (Supplementary Figure S5A), consistent with the biological heterogeneity of thyroid tumours. Importantly, RAIR scores retained the expected pathway association structure in the external cohort, showing positive correlations with hypoxia and interferon/inflammatory Hallmark programs (BH-FDR <0.05) and similar directional trends for EMT and angiogenesis (Supplementary Figure S5B–D).

We next performed pharmacology-oriented external analyses linked to the prioritised drug classes. In GSE126698, RAIR scores correlated with RAF–MEK–ERK axis components (e.g., SPRY2, MAPK1, MAP2K1 and RAF1; BH-FDR <0.05) and with VEGF/VEGFR-axis genes (most strongly TEK; Supplementary Figure S6B), and target-axis scores showed coherent variation across histologies (Supplementary Figure S6A). At the pathway level, RAIR scores were strongly associated with KRAS signaling up and PI3K–AKT–mTOR signaling (BH-FDR <0.01), and modestly with angiogenesis (BH-FDR <0.05; Supplementary Figure S6C). Together, these analyses provide independent external support that the RAIR transcriptional state aligns with drug-mechanism programs relevant to RAF/BRAF, VEGFR/KDR and PI3K–AKT–mTOR targeting.

### 3.4 RAIR signature stratifies TCGA tumours and transfers to DepMap models

To move from modules to patient-level readouts, we derived a single-sample RAIR signature by collapsing the RAIR-up module into a continuous score. For each TCGA-THCA tumour (n = 572), we z-standardised gene expression and averaged RAIR-up gene expression to obtain a RAIR score. The distribution was unimodal but right-skewed across tumours (Figure 1E). Using tertiles, we defined three strata—C1 (RAIR-low), C2 (RAIR-mid) and C3 (RAIR-high)—containing 191, 190 and 191 cases, respectively (Figure 4C; Table 3). Mean RAIR scores increased in a stepwise fashion from C1 to C3 (1.60 ± 0.09, 1.75 ± 0.03, 1.85 ± 0.03; Table 3), confirming that the tertiles capture a real gradient.

**TABLE 3:** Characteristics of TCGA RAIR tertile clusters.

Cluster	N	meanRAIRScore	sdRAIRScore	BRAFPercent	TERTPercent
C1 (RAIR_low)	191	1.604	0.093	55.50%	0.50%
C2 (RAIR_mid)	190	1.751	0.028	57.40%	0.50%
C3 (RAIR_high)	191	1.845	0.034	40.80%	0%

Summary statistics for RAIR tertile clusters in TCGA-THCA. For each cluster (C1, RAIR_low; C2, RAIR_mid; C3, RAIR_high), the table gives the number of tumours (N), the mean and standard deviation of the RAIR signature score (meanRAIRScore, sdRAIRScore), and the proportions of cases harbouring BRAF, mutations (BRAFPercent) and TERT promoter mutations (TERTPercent). The RAIR_high cluster shows the highest RAIR scores with only modest differences in driver mutation frequencies.

Principal component analysis (PCA) of Hallmark scores placed these tertiles along a continuum in pathway space: RAIR-high tumours clustered at one end of the first principal component, RAIR-low at the other, with RAIR-mid in between (Figure 4A). Correlating the RAIR score with individual Hallmarks further clarified its meaning. In TCGA, the score associated strongly with angiogenesis (r ≈ 0.83; Figure 4B), EMT (r ≈ 0.78; Figure 4D), G2M checkpoint, hypoxia and multiple inflammatory and interferon signatures (Table 2), consistent with the module-level enrichment in Section 3.2. In contrast, BRAF and TERT promoter mutations were not tightly aligned with RAIR strata: BRAF mutation frequencies were similar in C1 and C2 and only modestly lower in C3, and TERT mutations were rare in all three groups (Figures 4E,F; Table 3). Thus, the RAIR-high group reflects a composite pathway state rather than simply marking BRAF/TERT-mutant disease.

We then transferred the same signature to DepMap. Using the RAIR-up module, we computed a RAIR “cell score” (RAIR_score_cell in code) across 1,517 cell lines and examined its distribution. Globally, RAIR cell scores varied smoothly across lineages (Figures 5A,B,F), with fibroblast, lung and endocrine models tending towards higher scores and haematologic and central nervous system (CNS) models towards lower scores (Figure 5E). Within the 18 thyroid lines, scores spanned a considerable range but occupied a compact region in expression PCA space (Figure 5C). Dichotomising at the median split the thyroid panel into RAIR-low and RAIR-high models (Figure 5D; Supplementary Table S1). Importantly, in the thyroid subset the cell scores remained strongly correlated with Hallmark EMT, angiogenesis, inflammatory response, interferon-alpha/gamma, G2M checkpoint and apoptosis (Supplementary Figure S2A–G), mirroring the patient-level associations. Thus, a module-based RAIR signature robustly stratifies TCGA tumours and can be transplanted into cell-line models without losing its pathway-level interpretation, providing a mechanistic feature for downstream drug-response analyses.

Because the RAIR signature was derived from bulk transcriptomic data, we next examined whether it primarily reflected bulk composition rather than tumour-associated biology. In the discovery cohort GSE151179, RAIR and RAI-avid tumours did not show major differences in ESTIMATE-derived StromalScore, ImmuneScore, ESTIMATEScore or TumorPurity. We then projected the RAIR signature into TCGA-THCA and related it to tumour microenvironment-oriented features. The RAIR score showed a positive association with endothelial marker-based scores and a negative association with macrophage marker-based scores, whereas correlations with global ESTIMATE-derived purity and broad immune/stromal admixture metrics were weak. Together, these analyses argue against a simple bulk-purity explanation and instead suggest that the RAIR signature captures a composite programme that includes tumour-associated as well as selected microenvironment-linked signals (Supplementary Figure S8; Supplementary Table S3).

### 3.5 PRISM drug-response and multi-modal modelling in RAIR-like thyroid cell lines

We next characterised drug-response patterns in RAIR-like thyroid models using PRISM viability screens. In the PRISM dataset, 18 DepMap thyroid cell lines were profiled against ∼1,500 compounds, yielding 13,070 evaluable cell–drug AUC measurements (Supplementary Figure S1A–C). Most thyroid lines were assayed with over 1,000 drugs (Supplementary Figure S1A), and AUC values spanned a wide range with a long tail of highly potent agents (Supplementary Figure S1C). When compounds were stratified into targeted-like versus “other/unspecified” based on annotated mechanisms, targeted-like agents showed slightly lower median AUCs (Supplementary Figure S1E), suggesting that they are more likely to elicit strong viability reductions and therefore are natural candidates for RAIR-focused interrogation.

Using the RAIR cell scores to classify thyroid lines as RAIR-high or RAIR-low (Section 3.4), we defined for each drug a differential sensitivity ΔAUC = AUC_RAIR-high − AUC_RAIR-low. Negative ΔAUC denotes greater potency in RAIR-high lines, whereas positive values indicate relative resistance. This scalar summarises RAIR-selective response and is used both as a descriptive measure and as a target for machine-learning models and class-level aggregation.

To formally link cell state and drug identity to AUC, we trained regularised multi-modal regression models. For each thyroid cell–drug pair, we constructed three feature sets: (i) a cell-only representation using 16 expression-derived principal components; (ii) a drug-only representation using one-hot encoded drug identifiers; and (iii) a multi-modal representation concatenating cell features, including the RAIR cell score, with drug one-hot features. All models used L2-regularised linear regression (ridge) and were evaluated by five-fold cross-validation on PRISM AUC. The cell-only model had limited predictive power (r = 0.166, RMSE = 0.288; Figure 6A), indicating that subtle baseline transcriptional variation alone cannot explain most variance in AUC. The drug-only model captured a large fraction of the variance (r = 0.713, RMSE = 0.207; Figure 6B), reflecting intrinsic potency differences across drugs. Importantly, the multi-modal model integrating both cell and drug features further improved performance (r = 0.742, RMSE = 0.198; Figure 6C), providing a small but consistent gain over the drug-only baseline. This shows that RAIR-related transcriptional state contributes additional information about drug response that can be captured by a simple, interpretable machine-learning framework.

These results should be interpreted in the context of the modelling framework. Because PRISM responses are measured in cell-line monoculture systems, the multimodal model primarily captures tumour-cell-associated vulnerabilities rather than full patient-level drug efficacy. Consistent with this distinction, external validation in GSE126698 showed that RAIR scores remain associated with hypoxia and inflammatory/interferon programmes (Supplementary Figures S5–S6), while supplementary tumour-purity and microenvironment-oriented analyses suggested that the RAIR programme is not primarily explained by global bulk-purity differences but includes selected microenvironment-linked components (Supplementary Figure S8; Supplementary Table S3). Thus, tumour microenvironment-related processes are biologically relevant to the RAIR state, but are not directly encoded in the current predictor.

To determine whether this information was specific to the study-derived RAIR signature rather than a generic consequence of broader aggressive-state programmes, we benchmarked the RAIR signature against EMT, Hypoxia, Angiogenesis, Inflammatory Response and IFN-gamma signatures across public-cohort, drug-sensitivity and predictive settings. In GSE151179, the RAIR signature showed the strongest discrimination of RAIR versus RAI-avid tumours, supporting its disease-specific relevance. By contrast, transfer to four independent ATC public cohorts showed that the RAIR signature was not a generic ATC classifier, whereas several broader aggressive-state signatures, particularly hypoxia- and EMT-related programmes, showed more consistent ATC-high behaviour. In the DepMap/PRISM thyroid subset, however, the RAIR signature produced the most disease-aligned class-level shift for VEGFR/KDR inhibitors and yielded a clear positive gain when added to the drug-only baseline model. Hypoxia and EMT also provided non-trivial predictive improvements, indicating partial biological overlap between the RAIR programme and broader invasive-state signatures. Together, these analyses support interpreting the RAIR signature as a RAIR-focused transferable programme with disease-specific and partially non-redundant value, rather than as a uniformly dominant or pan-ATC signature (Supplementary Figure S9; Supplementary Table S4).

### 3.6 Class-level sensitivity and network linking RAIR biology to kinase inhibitors

We then leveraged ΔAUC to identify drugs and classes with preferential activity in RAIR-like thyroid models. For each targeted agent in the PRISM thyroid subset, we compared AUC distributions between RAIR-high and RAIR-low lines. Representative examples (Figures 7A–F) show that everolimus (mTOR inhibitor), regorafenib (multikinase), KI-8751 (VEGFR), BIBU-1361 (VEGFR/PDGFR) and telatinib (VEGFR) all display negative ΔAUC, with lower AUCs in RAIR-high lines, indicating greater sensitivity in RAIR-like states (Figures 7A–E). In contrast, dasatinib (SRC/ABL inhibitor) shows little or no RAIR-selective effect (Figure 7F). These cases illustrate that RAIR-like transcriptional states do not uniformly sensitise to all targeted agents, but certain VEGFR- and RAF-pathway drugs can exhibit clear RAIR-high–biased efficacy.

Across the entire targeted set, most compounds cluster around ΔAUC ≈0 and are not individually significant; however, a subset of agents, particularly among VEGFR/KDR and RAF/BRAF inhibitors, tend to have ΔAUC <0 (Supplementary Figure S4A). When drugs are grouped by class, the ΔAUC distributions (Supplementary Figure S4B) reveal that VEGFR/KDR and RAF/BRAF classes are skewed towards negative values, whereas RET and “Other/unspecified” classes are centred near zero. Class-level averaging confirms this pattern: VEGFR/KDR and RAF/BRAF inhibitors show the most negative mean ΔAUC (−0.096 and −0.193, respectively), with mTOR inhibitors showing a weaker but consistent negative shift (mean ΔAUC ≈ −0.029), while other classes are near neutral (Figure 8A; Supplementary Table S2). Class sizes range from a few to dozens of drugs, with some classes (such as mTOR inhibitors) comprising more than one hundred compounds (Figure 8B), arguing against single-drug outliers as the sole driver. Within VEGFR/KDR and RAF/BRAF classes, per-drug dot plots (Figures 8C,D) highlight individual inhibitors with particularly strong RAIR-high selectivity, helping to prioritise candidates within broadly active families.

Finally, we integrated transcriptional modules, pathway activities, drug classes and RAIR-high models into a unified network (Figure 9). The RAIR module node connects to EMT, angiogenesis, inflammatory, interferon and G2M Hallmarks, recapitulating the RAIR–ATC axis defined in Sections 3.2, 3.3. These Hallmarks in turn link to VEGFR/KDR and RAF/BRAF inhibitor classes whose experimental class-level ΔAUC and multi-modal predictions both indicate preferential activity in RAIR-high thyroid cells. Edges from drug classes to individual RAIR-high lines encode relative sensitivity derived from ΔAUC and modelled AUC, thereby nominating specific experimental models for further validation. Together, the class-level analysis and integrative network connect RAIR biology to concrete targeted-therapy hypotheses, prioritising VEGFR/KDR and RAF/BRAF inhibitor classes as candidates for further translational evaluation in RAIR-like thyroid cancer states.

### 3.7 *In vitro* validation of predicted sensitivity to VEGFR/RAF pathway inhibition

To obtain initial functional support for the computationally predicted RAIR-associated vulnerabilities, we performed proof-of-concept *in vitro* validation in the thyroid cancer cell line CAL-62, selected as a RAIR-high/ATC-like model based on its high RAIR score in our DepMap transcriptomic analyses. Sorafenib was prioritised for validation because it targets kinases central to the inferred RAIR axis and PRISM-derived drug-class hypotheses, including RAF-family kinases and angiogenesis-related receptor tyrosine kinases.

**FIGURE 10:**
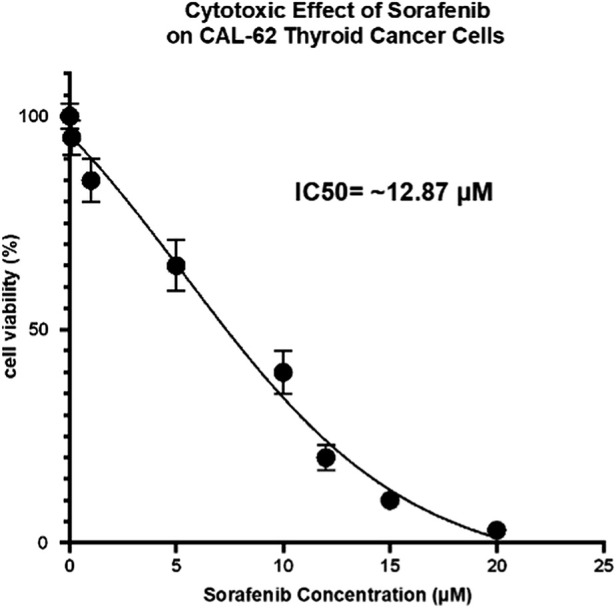
*In vitro* validation of predicted sensitivity to sorafenib in a RAIR-high thyroid cancer cell line. CAL-62 thyroid cancer cells, selected as a RAIR-high/ATC-like model based on DepMap transcriptomic analyses, were treated with increasing concentrations of sorafenib for 72 h. Cell viability was measured using a MTT assay and normalised to vehicle control. Sorafenib reduced cell viability in a dose-dependent manner, with an estimated IC50 of approximately 12.9 µM. Data are shown as mean ± SD of three technical replicates from a representative experiment; similar results were observed in at least three independent experiments.

Cell viability assay. CAL-62 cells were seeded in 96-well plates (3,000 cells/well) and treated with a log-spaced concentration series of sorafenib (10-point dilution spanning 0.03–30 µM) with vehicle (DMSO) controls. After 72 h of exposure, cell viability was quantified using an MTT assay and normalised to vehicle-treated controls. Sorafenib induced a clear dose-dependent reduction in viability (Figure 10). Nonlinear dose–response modelling (four-parameter logistic fit) yielded an estimated IC50 ≈ 12.9 µM, indicating substantial sensitivity in this RAIR-like model. Data are presented as mean ± SD of three technical replicates, and the experiment was repeated in at least three independent runs with consistent results.

**FIGURE 11:**
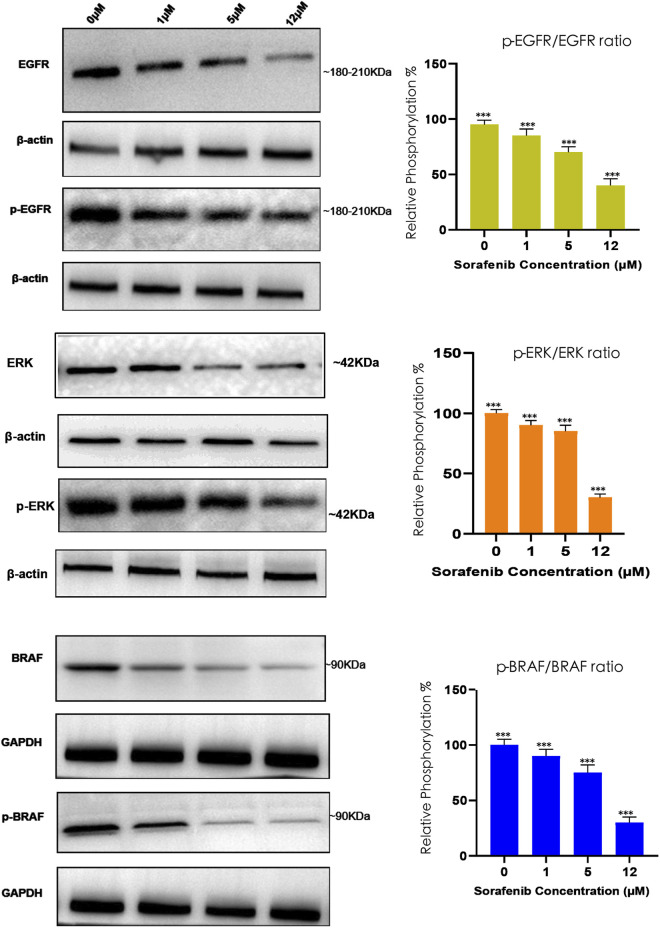
Sorafenib reduces EGFR, ERK and BRAF phosphorylation in RAIR-high thyroid cancer cells. CAL-62 cells were treated with sorafenib (0, 1, 5 and 12 µM) for 6 h. Representative immunoblots show total and phosphorylated EGFR, ERK and BRAF, with β-actin or GAPDH used as loading controls. Right panels show densitometric quantification of the p-EGFR/EGFR, p-ERK/ERK and p-BRAF/BRAF ratios, normalised to the 0 µM control. These results provide proof-of-concept biological support for the predicted vulnerability of the RAIR-high/ATC-like CAL-62 model to VEGFR/RAF pathway inhibition. Uncropped immunoblots are provided in Supplementary Figure S7. Values represent mean ± SD from three independent experiments. **P < 0.01, ***P < 0.001 versus 0 µM control by one-way ANOVA with Dunnett’s *post hoc* test.

Pathway inhibition by immunoblotting. To verify that the observed cytotoxicity was accompanied by suppression of the predicted signaling programs, we assessed pathway activity following sorafenib treatment by immunoblotting (Figure 11). CAL-62 cells were treated with sorafenib (0, 1, 5 and 12 µM) for 6 h, lysed, and analysed for upstream receptor tyrosine kinase signalling and downstream RAF–MAPK pathway activity. Sorafenib induced dose-dependent reductions in the p-EGFR/EGFR, p-ERK/ERK and p-BRAF/BRAF ratios, with the strongest suppression observed at 12 µM (Figure 11). β-Actin or GAPDH served as loading controls, and densitometric quantification confirmed a progressive decrease in pathway phosphorylation with increasing sorafenib concentration.

Together, these *in vitro* results provide initial proof-of-concept support for the predicted vulnerability of a RAIR-high/ATC-like thyroid model to VEGFR/RAF pathway inhibition, as inferred from PRISM differential-response analyses and multimodal modelling, and complement the external mechanism-oriented validation in GSE126698 (Supplementary Figures S5–S6).

## 4 Discussion

Radioiodine-refractory papillary thyroid carcinoma (RAIR PTC) and anaplastic thyroid carcinoma (ATC) represent the clinically most challenging ends of the thyroid cancer spectrum (Haugen et al., 2016; Karapanou et al., 2022; Chen et al., 2024; Subbiah et al., 2022; Cabanillas et al., 2024). Yet, the molecular relationship between radioiodine refractoriness and anaplastic transformation has remained incompletely characterised, and it has been unclear whether drug-sensitivity patterns in RAIR-like models can be anticipated from transcriptional information (Sharifi-Noghabi et al., 2021; Firoozbakht et al., 2022; Chawla et al., 2022; Jiang and Li, 2024; Zhou et al., 2025). In this study, we address both questions by integrating multi-cohort bulk transcriptomes, DepMap cell-line data and PRISM drug-response profiles within a unified, module-based and machine-learning–enabled framework.

At the gene-expression level, differential analyses in an RAIR PTC cohort and five independent ATC cohorts revealed extensive and partially overlapping transcriptional reprogramming (Hebrant et al., 2012; Colombo et al., 2020; Yu et al., 2016; Hu et al., 2018; Wu et al., 2024). Aggregating recurrently altered genes into RAIR and ATC up- and downregulated modules (RAIR-up, RAIR-down, ATC-up and ATC-down) allowed us to move beyond cohort-specific gene lists and to focus on reproducible programmes. Functional annotation of these modules against Hallmark gene sets showed a striking convergence on a relatively small set of pathways. Both RAIR-up and ATC-up modules were enriched for epithelial–mesenchymal transition, angiogenesis, inflammatory and interferon responses, and G2/M checkpoint and mitotic control, whereas differences in canonical PI3K–AKT–mTOR signalling were comparatively subtle. ATC generally showed stronger enrichment, consistent with ATC representing a more extreme manifestation of the same underlying axis. These observations support the view that RAIR disease and anaplastic transformation occupy different positions on a shared EMT–angiogenesis–inflammatory trajectory rather than representing unrelated end states. We interpret this broader RAIR–ATC framework as a transcriptional continuum, whereas the study-derived RAIR signature should be viewed as a disease-focused readout of this continuum rather than a discrete subtype label or a generic ATC classifier.

To translate this axis into a patient-level readout, we collapsed the RAIR-up module into a single-sample score by averaging z-normalised expression of module genes. In the TCGA cohort, the resulting RAIR score spanned a broad range and aligned with a dominant principal component of Hallmark activity, indicating that it captures a major mode of transcriptomic variation. Stratification by tertiles yielded three RAIR strata with stepwise increases in mean score and clear, albeit continuous, separation in Hallmark principal-component space. Correlation analyses showed that high RAIR scores were tightly associated with activation of epithelial–mesenchymal transition, angiogenesis, cell-cycle progression, hypoxia and multiple inflammatory and interferon signatures, mirroring the module-level enrichments. Importantly, these strata were not simply surrogates for BRAF or TERT promoter mutation status: mutation frequencies were similar or only modestly different across RAIR-low, intermediate and high groups, and TERT promoter mutations were rare overall. Thus, the RAIR score appears to capture a multi-gene functional state that sits downstream or parallel to canonical drivers, emphasising pathway-level dysregulation rather than single-gene events.

Biologically, the association of the RAIR state with EMT, angiogenesis, hypoxia and inflammatory/interferon programmes is consistent with the dedifferentiated phenotype of radioiodine-refractory thyroid cancer. Loss of iodine avidity is unlikely to be a purely single-gene event and may instead accompany broader transcriptional reprogramming involving reduced thyroid-lineage identity, increased mesenchymal features, vascular remodelling and inflammatory stress signalling. In this context, the RAIR signature may capture a state in which impaired radioiodine handling coexists with more aggressive and therapy-relevant pathway dependencies, rather than reflecting radioiodine resistance as an isolated molecular event.

We then asked whether this signature retains its biological meaning in experimental models. Projecting the RAIR-up module onto DepMap transcriptomes produced a per-cell RAIR score that varied smoothly across tumour lineages. Thyroid cell lines formed a compact cluster in expression space but spanned a substantial range of RAIR scores, and a simple median split separated them into RAIR-low and RAIR-high groups that were not confined to a single histology. Across all DepMap lineages, the RAIR score showed an approximately normal distribution with lineage-specific shifts, indicating that the underlying axis is not unique to thyroid cancer but is particularly relevant there. Critically, in thyroid cell lines the RAIR score remained correlated with the same Hallmark programmes—epithelial–mesenchymal transition, angiogenesis, inflammatory and interferon responses, cell-cycle control and apoptosis—that define the RAIR axis in patients. This preservation of pathway context suggests that a substantial tumour-associated component of the RAIR programme is retained in cell-line models, although contributions from microenvironment-linked signals cannot be excluded because the original signature was derived from bulk tumour transcriptomes.

Importantly, the additional benchmark analyses refine how this signature should be interpreted. Although the broader RAIR–ATC axis overlaps with aggressive programmes that are also captured by EMT-, hypoxia- and inflammation-related signatures, the study-derived RAIR signature showed the strongest specificity for the clinically relevant RAIR-versus-RAI-avid contrast and provided disease-focused value in VEGFR/KDR-related vulnerability prioritisation. At the same time, its transfer to independent ATC cohorts was not uniformly ATC-high, indicating that it should not be interpreted as a generic ATC classifier. We therefore view the RAIR signature as a RAIR-focused transferable programme that partially overlaps with, but is not equivalent to, broader ATC-associated aggressive transcriptional states (Supplementary Figure S9; Supplementary Table S4).

The PRISM viability screens provide an unprecedented opportunity to link such transcriptional states to small-molecule sensitivity at scale. In the thyroid subset, 18 DepMap cell lines were profiled against about 1,500 compounds, yielding more than 13,000 AUC measurements. To summarise RAIR-selective response, we defined for each drug a simple differential metric, the difference in AUC between RAIR-high and RAIR-low cell lines. Negative values indicate higher potency in RAIR-high models. This ΔAUC captures RAIR-biased activity in a single number and serves as a common phenotype for exploratory plots, class-level summaries and model evaluation.

On top of these data, we built regularised linear models that integrate transcriptional and drug-level information (Sharifi-Noghabi et al., 2021; Firoozbakht et al., 2022; Chawla et al., 2022; Jiang and Li, 2024; Zhou et al., 2025). Three configurations were considered: a cell-only variant based on principal components of gene expression, a drug-only variant based on one-hot encoded compound identifiers, and a multi-modal variant that concatenates both feature sets together with the RAIR score. All models were trained to predict AUC using five-fold cross-validation. As expected, the cell-only model had limited predictive power, reflecting the small number of thyroid lines and the modest dynamic range of transcriptional variation. Drug identity alone accounted for a large fraction of explainable variance, underlining the dominant influence of intrinsic potency differences. Importantly, however, the multi-modal model consistently outperformed the drug-only baseline in terms of correlation and error, demonstrating that RAIR-related expression features provide non-redundant information about response. From a methodological perspective, this shows that even relatively simple, interpretable machine-learning models can leverage biologically meaningful signatures to refine drug-sensitivity predictions in small panels.

The differential-response metric and the multi-modal predictions were then aggregated at the class level to obtain a higher-level view of RAIR-selective sensitivity. Across targeted mechanisms, VEGFR/KDR and RAF/BRAF inhibitors displayed the most negative class-averaged ΔAUC values, indicating greater potency in RAIR-high relative to RAIR-low cell lines. mTOR inhibitors also showed a weaker but consistent negative shift, whereas RET and other classes exhibited little directional bias. Within the VEGFR/KDR and RAF/BRAF classes, several individual inhibitors stood out as particularly RAIR-selective, suggesting concrete candidates for further prioritisation. Although the effect sizes are moderate and the number of models limited, the fact that these classes emerge as outliers against a backdrop of largely neutral ΔAUC distributions supports the prioritisation of vascular and RAF-pathway targeting as candidate therapeutic directions in RAIR-like states. Mechanism-oriented analyses in an independent RNA-seq cohort (GSE126698) further supported that higher RAIR scores are associated with increased RAF–MEK–ERK and VEGF/VEGFR axis activity, as well as KRAS and PI3K–AKT–mTOR programs (Supplementary Figures S5–S6), consistent with the drug-class hypotheses derived from PRISM. Importantly, we further complemented these *in silico* findings with direct *in vitro* validation: sorafenib elicited dose-dependent cytotoxicity in the RAIR-high/ATC-like CAL-62 model and suppressed RAF–MAPK pathway activation (Figures 10, 11), providing biological support for the predicted vulnerability to VEGFR/RAF pathway inhibition.

To synthesise these multi-layered findings, we constructed a compact network in which the RAIR module connects to its key Hallmark programmes, those programmes connect to drug classes with negative class-level ΔAUC and favourable model predictions, and the drug classes connect to RAIR-high thyroid cell lines that could serve as experimental validation systems. This graph highlights a coherent path from RAIR transcriptional biology, through EMT and angiogenesis and inflammatory signalling, to enhanced sensitivity to VEGFR/KDR and RAF/BRAF inhibition in specific RAIR-like models. Conceptually, it closes the loop between discovery in patient cohorts, transfer to cell lines, machine-learning–guided interpretation of drug response and concrete therapeutic hypotheses.

### 4.1 Several limitations merit discussion

First, the RAIR discovery cohort is relatively small and clinically heterogeneous, and radioiodine refractoriness itself is a composite phenotype influenced by tumour burden, uptake kinetics and treatment history (Karapanou et al., 2022; Volpe et al., 2024; Schubert et al., 2024). Second, because the RAIR signature was derived from bulk transcriptomic data, it may reflect both tumour-cell-associated and microenvironment-linked signals. Our supplementary tumour-purity and tumour microenvironment-oriented analyses did not support major confounding by global bulk purity, but did suggest associations with endothelial- and macrophage-related signals (Supplementary Figure S8; Supplementary Table S3). We therefore interpret the RAIR signature as a composite state measure rather than a purely tumour-cell-intrinsic programme, and future single-cell or spatial studies will be needed to resolve its cellular origin more precisely. Third, our multimodal predictor was trained on DepMap and PRISM cell-line data and thus models drug response in a simplified tumour-cell-centred system without explicit patient-level tumour microenvironment variables such as immune infiltration, stromal composition or hypoxia severity. This is particularly relevant for anti-angiogenic and pathway-targeted agents, whose efficacy *in vivo* may be influenced by vascular context, paracrine signalling and drug exposure; accordingly, we regard the current framework as a translational prioritisation tool for RAIR-like intrinsic vulnerabilities rather than a direct clinical response model. Fourth, functional validation was limited to a single RAIR-high/ATC-like thyroid cancer cell line (CAL-62), so the current *in vitro* results should be viewed as proof-of-concept support rather than definitive evidence of generalisability across RAIR-like thyroid cancers. Fifth, the machine-learning models were evaluated by internal cross-validation only, and broader validation on independent screens or prospective experiments will be required. Likewise, external validation in GSE126698 mainly supports pathway- and mechanism-level transferability of the RAIR signature across platforms, rather than robust histological discrimination, which is likely constrained by cohort size and tumour heterogeneity.

Despite these caveats, the overall picture is internally consistent. The RAIR signature derived from a small but carefully annotated cohort aligns with the dominant transcriptomic variation in TCGA, retains its pathway associations in cell-line models, and contributes incremental predictive value beyond drug identity in multi-modal regression. Drug- and class-level differential AUC analyses converge on VEGFR/KDR and RAF/BRAF inhibitors as the most promising RAIR-selective classes, and the integrative network shows how these classes are mechanistically linked to the underlying EMT–angiogenesis–inflammatory axis.

Future work should extend this framework in several directions. At the biological level, single-cell and spatial transcriptomics in RAIR and ATC tumours could refine the cellular origin of the RAIR signature and disentangle tumour-intrinsic from microenvironmental components. Functionally, CRISPR or pharmacological perturbations in RAIR-high versus RAIR-low cell lines could test causality between EMT/angiogenesis programmes and drug sensitivity. On the modelling side, more expressive architectures (for example, graph neural networks over drug structures or non-linear multi-task models across tumour types) may further enhance predictive power and generalisability. Ultimately, prospective clinical studies will be needed to determine whether RAIR signature–guided selection of VEGFR/KDR or RAF/BRAF inhibitors improves outcomes for patients with RAIR or ATC-like thyroid cancers.

## 5 Conclusion

In summary, we used a module-based, multi-cohort framework to link radioiodine refractoriness, anaplastic transformation and targeted drug sensitivity in thyroid cancer. RAIR and ATC expression modules converged on a shared EMT–angiogenesis–inflammatory axis, from which we derived a single-sample RAIR signature that stratifies TCGA-THCA tumours and remains largely independent of BRAF/TERT status. Projection of this signature onto DepMap transcriptomes yielded RAIR cell scores that preserved the same pathway context in thyroid cell lines and supported a RAIR-high versus RAIR-low definition for experimental models. External RNA-seq validation and pharmacology-oriented analyses further supported the transferability of the signature and its alignment with drug-mechanism programs relevant to targeted anti-cancer drugs. Coupling these models to PRISM drug-response data, and embedding RAIR-related features into a multimodal ridge model, highlighted VEGFR/KDR and RAF/BRAF inhibitor classes as candidates for further translational evaluation in RAIR-like thyroid states. Overall, the additional benchmark analyses indicate that this RAIR signature is best interpreted as a disease-focused and partially non-redundant programme for RAIR-specific discrimination and therapeutic prioritisation, rather than a universally superior or pan-ATC signature.

## Data Availability

Publicly available datasets were analyzed in this study. This data can be found here: TCGA-THCA RNA-seq, clinical and mutation data were obtained from The Cancer Genome Atlas via the Genomic Data Commons (TCGA-THCA project, https://portal.gdc.cancer.gov/projects/TCGA-THCA) and UCSC Xena (https://xenabrowser.net). RAIR PTC and ATC expression cohorts were downloaded from the NCBI Gene Expression Omnibus (GSE151179, GSE27155, GSE33630, GSE53072, GSE65144 and GSE85457; e.g., https://www.ncbi.nlm.nih.gov/geo/query/acc.cgi?acc=GSE151179). DepMap expression profiles, cell-line annotations and PRISM secondary screen AUC values for thyroid cell lines were retrieved from the Broad DepMap portal (DepMap Public release, https://depmap.org/portal/, including PRISM data at https://depmap.org/portal/prism/). Additional drug-sensitivity information was obtained from the Genomics of Drug Sensitivity in Cancer (GDSC) resource (https://www.cancerrxgene.org/). All analysis scripts used in this study (including data preprocessing, RAIR/ATC module construction, RAIR signature and RAIR cell score calculation, PRISM Delta AUC analysis, and multimodal ridge modelling) are available at our GitHub repository: https://github.com/LiutianyuMedical/Thyroid-Medical.
