# Supplementary material for: A RAIR–ATC transcriptional axis and multimodal drug-response modelling reveal class-level vulnerabilities in thyroid cancer
Source: Front Pharmacol. 2026 May 18;17:1789743. doi: 10.3389/fphar.2026.1789743 (PMC13223037; doi:10.3389/fphar.2026.1789743)
Supplement: Supplementary file 1 [file Supplementaryfile1.pdf]

Supplementary

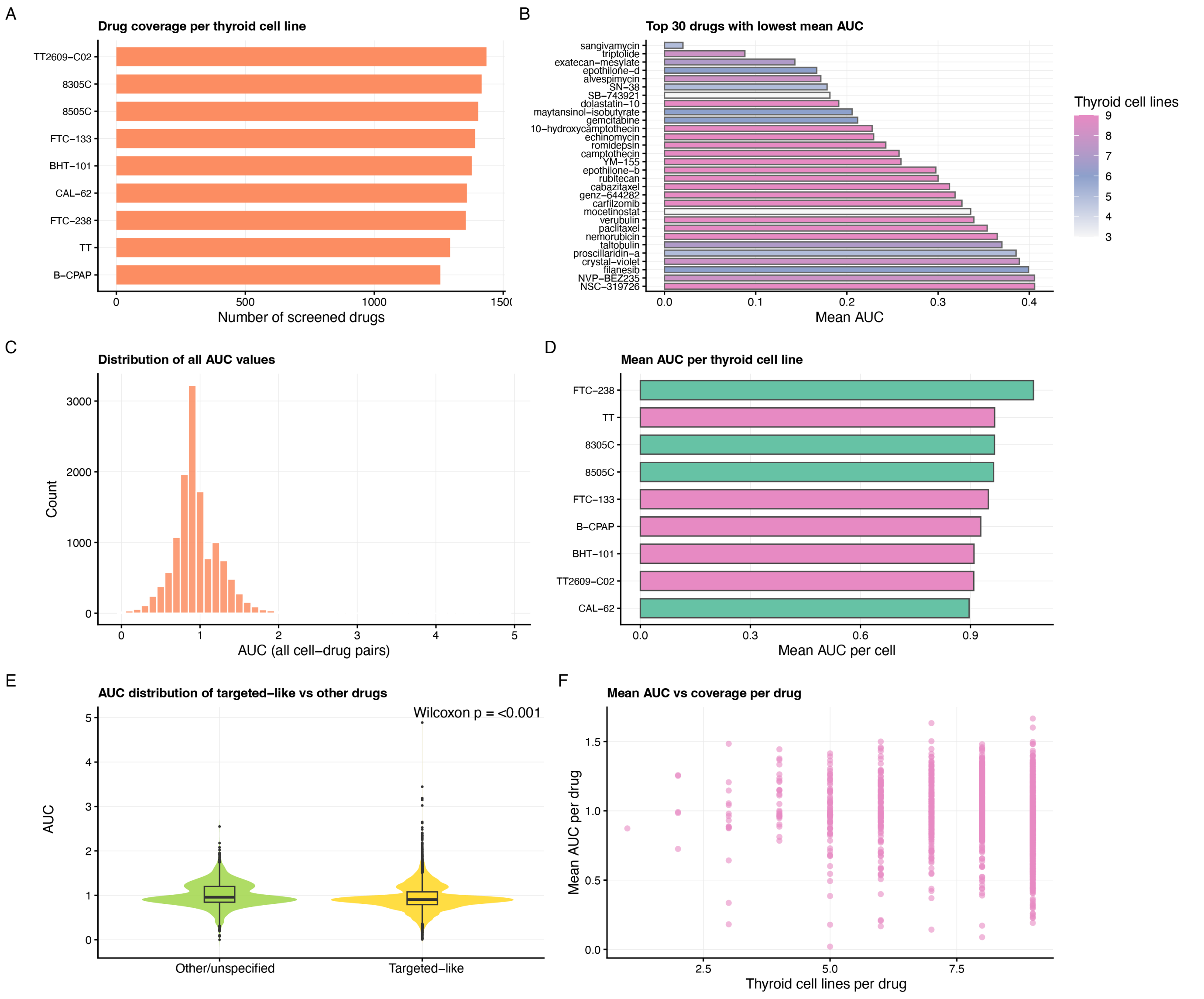

Figure S1. PRISM drug response landscape in thyroid cell lines.

(A) Number of screened drugs per thyroid cell line in the PRISM dataset, showing that most models are profiled with over 1,000 compounds.

(B) Top 30 compounds with the lowest mean AUC across thyroid cell lines; bars are coloured by the number of lines tested per compound.

(C) Distribution of AUC values for all thyroid cell–drug pairs, illustrating the overall response range in the PRISM thyroid subset.

(D) Mean AUC per thyroid cell line, highlighting global differences in baseline drug sensitivity between models.

(E) Distribution of AUC values for targeted-like versus other/unspecified compounds; the p-value from a Wilcoxon rank-sum test is shown.

(F) Relationship between mean AUC per drug and the number of thyroid cell lines tested for that drug, indicating that coverage does not trivially drive class-level sensitivity patterns.

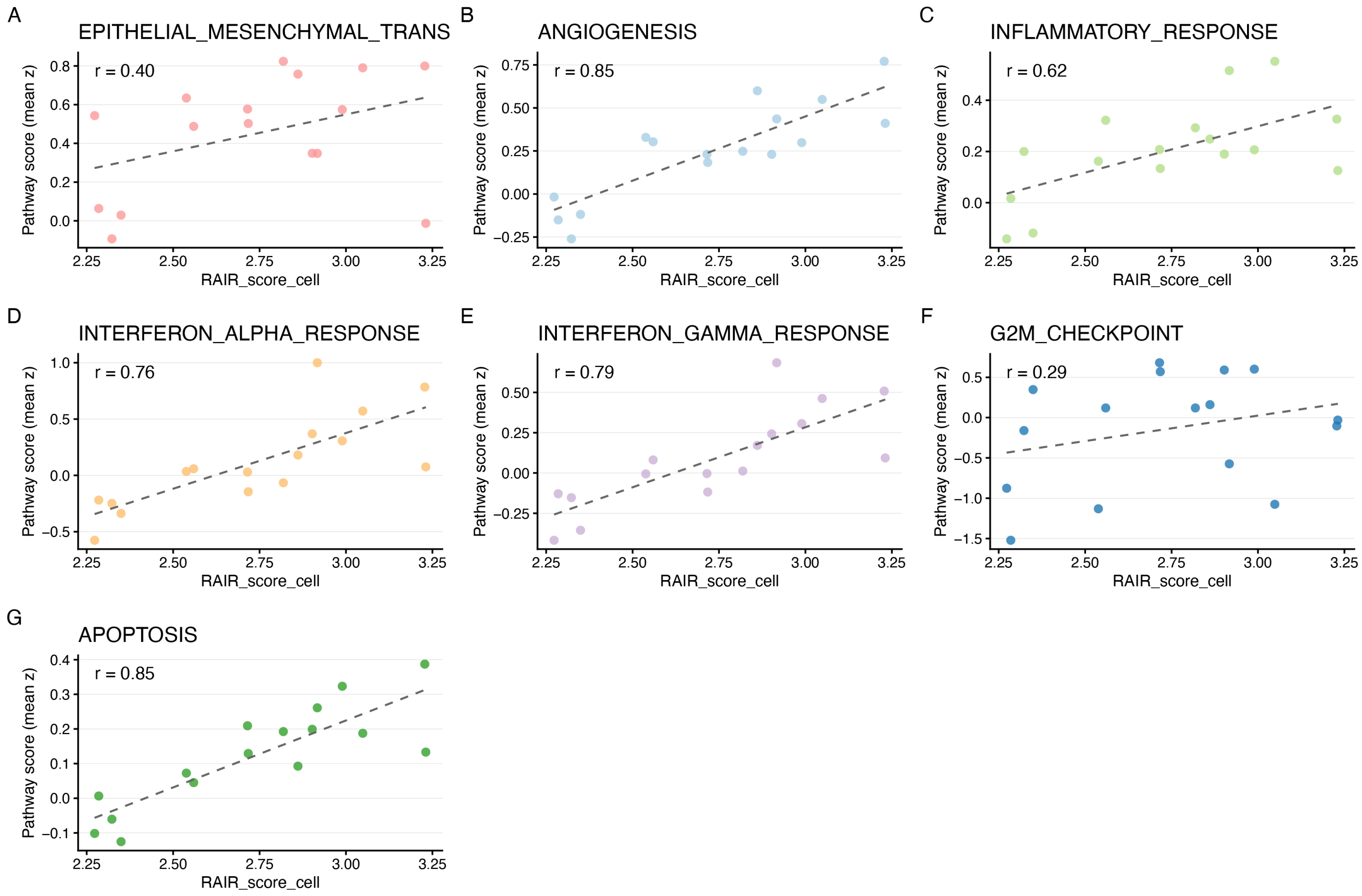

Figure S2. Association between RAIR_score_cell and Hallmark pathways in DepMap thyroid models. Scatter plots of RAIR_score_cell versus Hallmark pathway scores (mean z-score) for DepMap thyroid cell lines. Each panel shows the Pearson correlation coefficient (r) and a fitted linear regression line.

(A) EPITHELIAL_MESENCHYMAL_TRANSITION.
(B) ANGIOGENESIS.
(C) INFLAMMATORY_RESPONSE.
(D) INTERFERON_ALPHA_RESPONSE.
(E) INTERFERON_GAMMA_RESPONSE.
(F) G2M_CHECKPOINT.
(G) APOPTOSIS.

These associations recapitulate the key RAIR-related pathways identified in TCGA and RAIR/ATC modules, supporting the use of RAIR_score_cell as a pathway-level surrogate in experimental models.

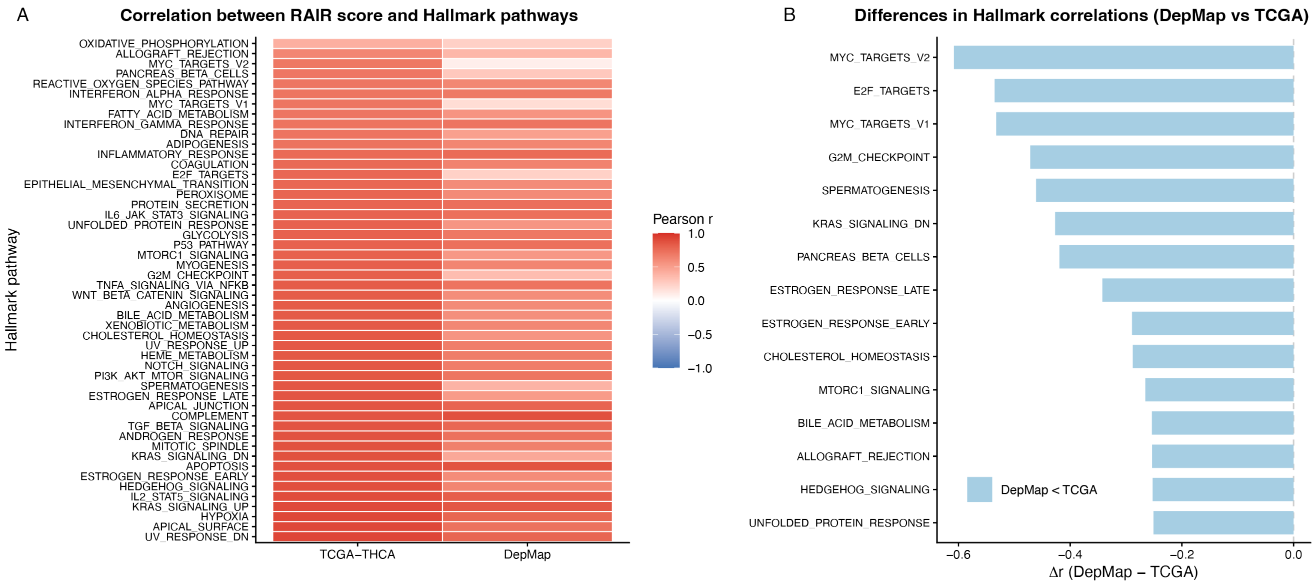

Figure S3. Global correlation of RAIR signature with Hallmark pathways in TCGA and DepMap.
(A) Heatmap of Pearson correlation coefficients between the RAIR signature score and 50 Hallmark pathways in TCGA-THCA tumours (left column) and DepMap cell lines (right column). Most pathways show positive correlations in both datasets, including interferon responses, inflammatory response, EMT, angiogenesis and G2M checkpoint.
(B) Differences in correlation strength between DepMap and TCGA (Δr = r_DepMap − r_TCGA) for the top pathways. Bars to the left indicate pathways where the correlation is weaker in cell lines than in tumours. Overall, the majority of RAIR-associated pathways show consistent directionality across patient samples and DepMap cell lines.

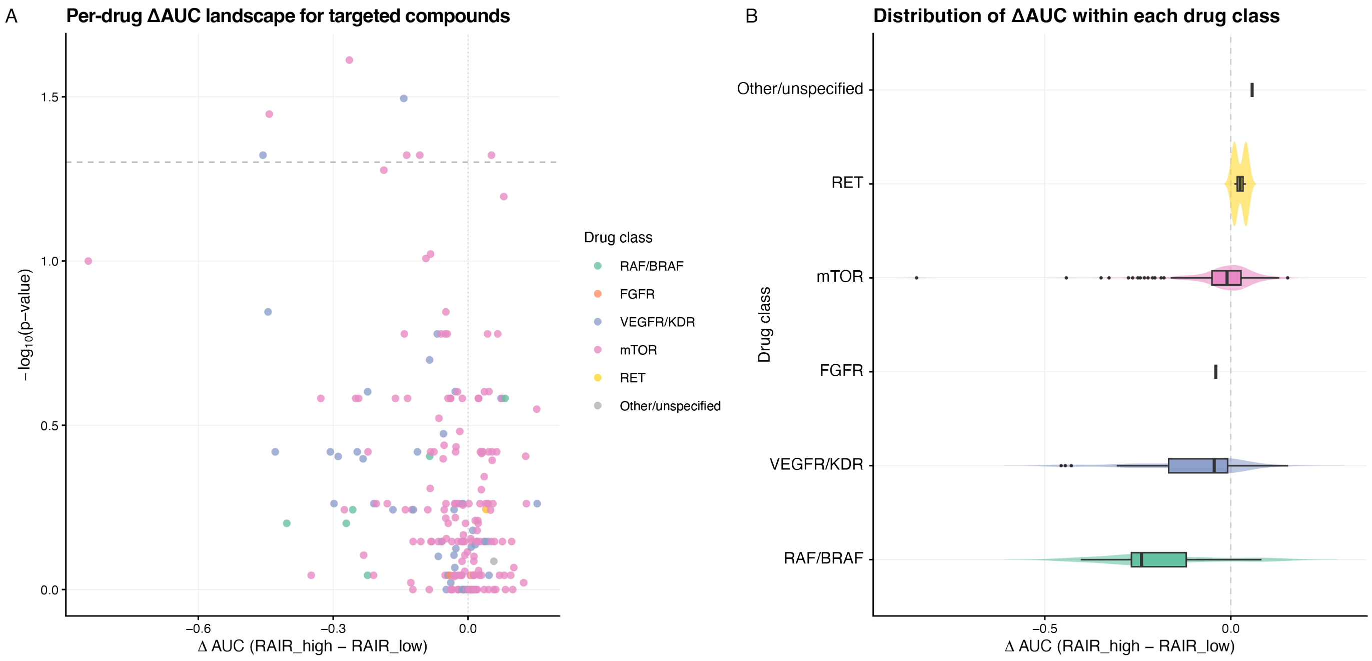

Figure S4. Per-drug Delta AUC landscape for targeted compounds.
(A) Per-drug Delta AUC (RAIR_high − RAIR_low) versus $-\log_{10}$(p-value) for all targeted compounds in the PRISM thyroid subset. Each point represents a drug and is coloured by its annotated drug class (RAF/BRAF, FGFR, VEGFR/KDR, mTOR, RET, Other/unspecified). The vertical dashed line denotes zero Delta AUC; the horizontal line marks the nominal significance threshold.
(B) Distribution of per-drug Delta AUC within each targeted class, shown as violin and box plots. Negative values indicate greater sensitivity (lower AUC) in RAIR_high cells. RAF/BRAF and VEGFR/KDR inhibitors are enriched for drugs with more negative Delta AUC, whereas mTOR inhibitors show a milder shift and other classes are largely centred near zero.

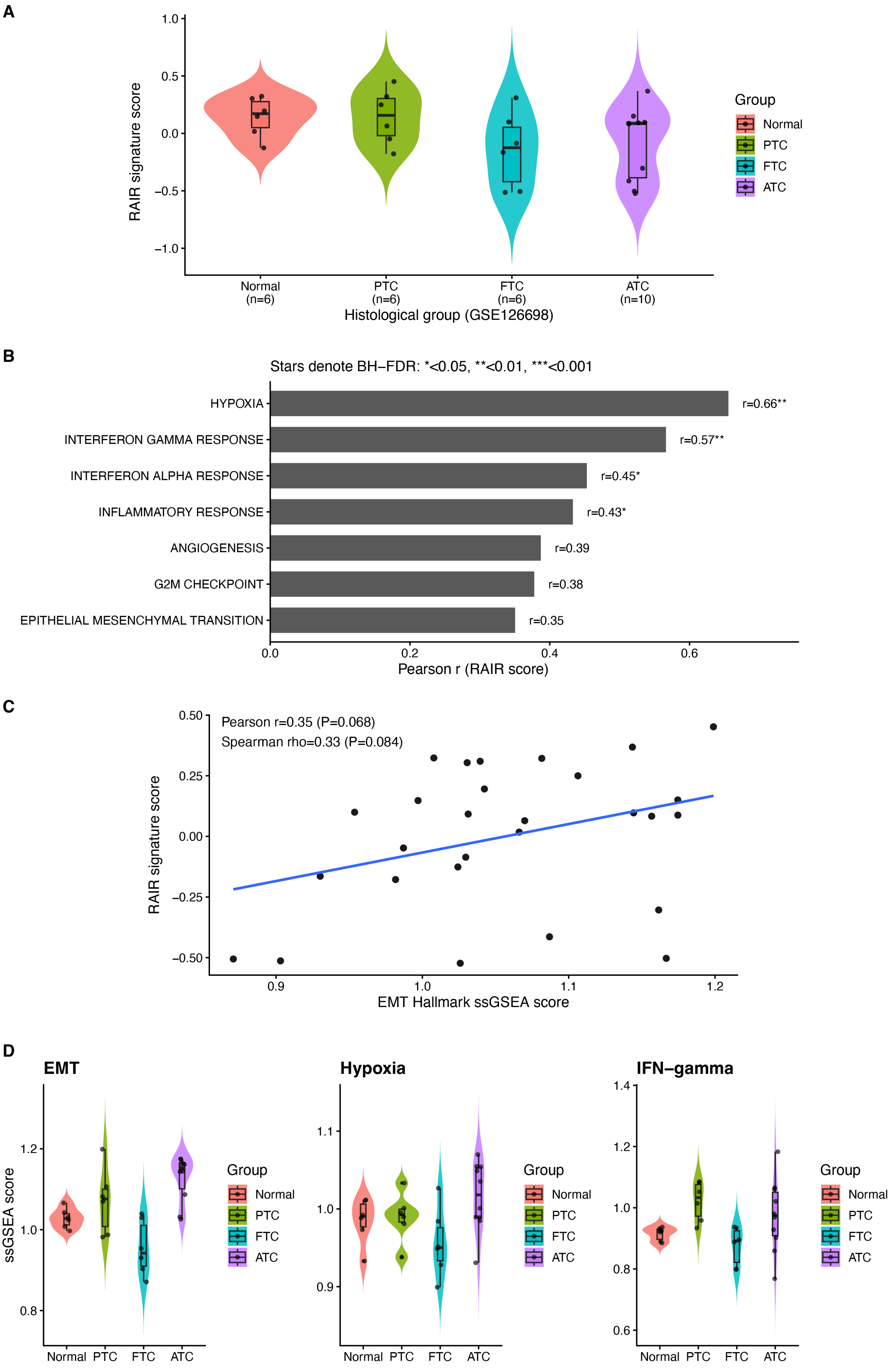
A
RAIR signature score
1.0
0.5
0.0
−0.5
−1.0
Normal (n=6)
PTC (n=6)
FTC (n=6)
ATC (n=10)
Histological group (GSE126698)
Group
Normal
PTC
FTC
ATC
B
Stars denote BH−FDR: *<0.05, **<0.01, ***<0.001
HYPOXIA
INTERFERON GAMMA RESPONSE
INTERFERON ALPHA RESPONSE
INFLAMMATORY RESPONSE
ANGIOGENESIS
G2M CHECKPOINT
EPITHELIAL MESENCHYMAL TRANSITION
r=0.66**
r=0.57**
r=0.45*
r=0.43*
r=0.39
r=0.38
r=0.35
0.0
0.2
0.4
0.6
Pearson r (RAIR score)
C
Pearson r=0.35 (P=0.068)
Spearman rho=0.33 (P=0.084)
RAIR signature score
0.50
0.25
0.00
−0.25
−0.50
0.9
1.0
1.1
1.2
EMT Hallmark ssGSEA score
D
EMT
Hypoxia
IFN−gamma
ssGSEA score
1.2
1.0
0.8
1.1
1.0
0.9
1.4
1.2
1.0
0.8
0.6
Normal
PTC
FTC
ATC
Group
Normal
PTC
FTC
ATC

Figure S5. Independent validation of the RAIR signature in an external RNA-seq cohort (GSE126698).
(A) RAIR signature scores across normal thyroid tissue (Normal), papillary thyroid carcinoma (PTC), follicular thyroid carcinoma (FTC), and anaplastic thyroid carcinoma (ATC). RAIR scores were computed using the same 300-gene RAIR-up module as in the main analyses after log1p transformation and gene-wise z-standardisation, followed by averaging z-scores within each sample. Violin plots show score distributions with embedded boxplots and individual samples overlaid; group sample sizes are indicated beneath each category.
(B) Pearson correlations between RAIR scores and selected MSigDB Hallmark pathway activities computed by ssGSEA (GSVA) in GSE126698. Bars denote correlation coefficients; asterisks indicate BH-FDR significance (*FDR<0.05, **FDR<0.01, ***FDR<0.001).
(C) Scatter plot showing the relationship between RAIR score and EMT Hallmark ssGSEA score in the external cohort. The fitted line represents a linear regression; Pearson and Spearman correlation coefficients (and P values) are displayed.
(D) ssGSEA activity scores for EMT, hypoxia and interferon-gamma Hallmark programs across histological groups in GSE126698, shown as violin/box plots with individual samples overlaid.

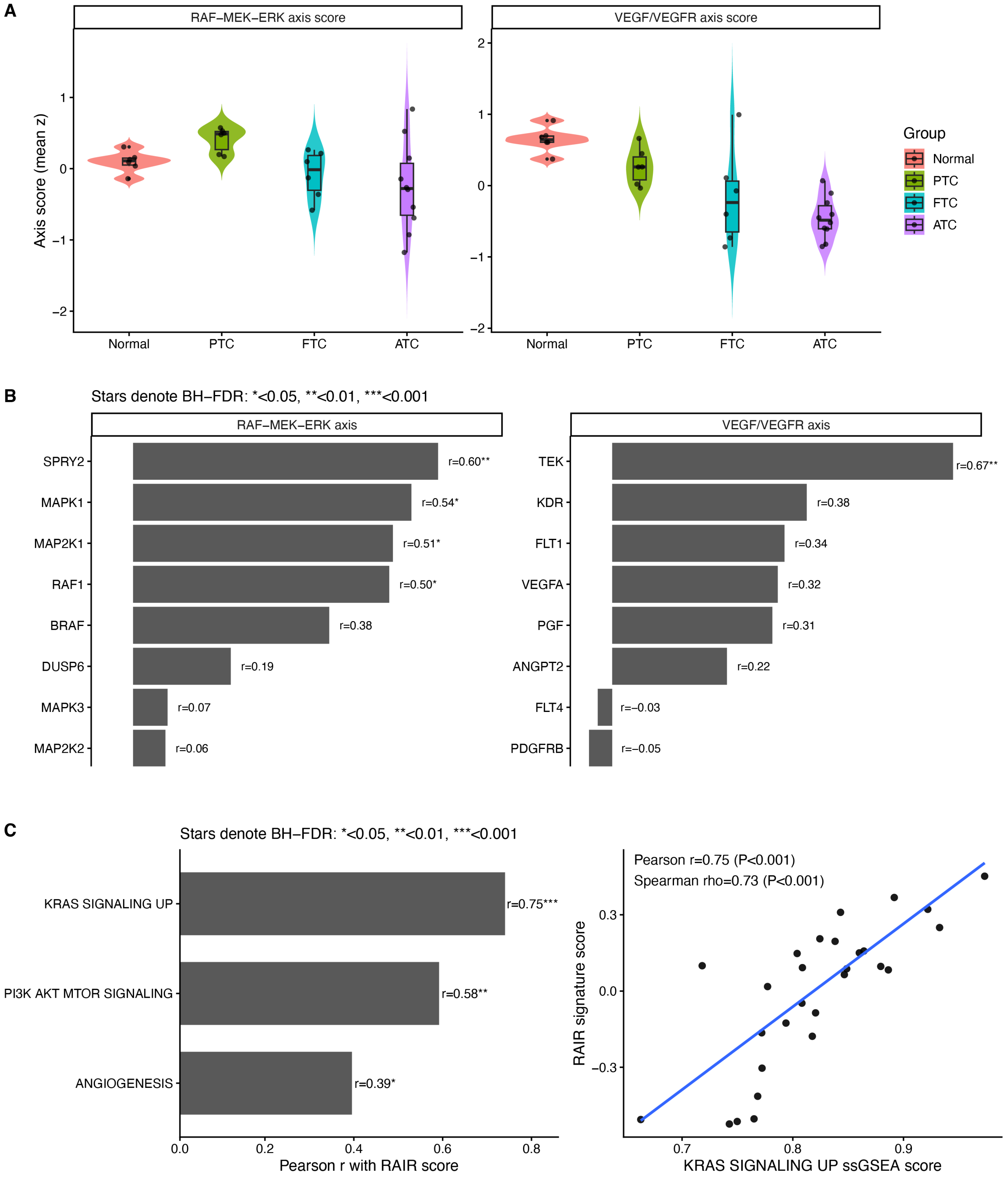

Figure S6. Pharmacology-oriented external support for candidate targeted drug classes in GSE126698. (A) Target-axis scores across histological groups. RAF–MEK–ERK and VEGF/VEGFR axis scores were computed as the mean gene-wise z-score across predefined axis gene sets (log1p-transformed expression), and are shown as violin/box plots with individual samples overlaid for Normal, PTC, FTC and ATC samples in GSE126698.

(B) Correlations between target-axis gene expression and RAIR signature score in the external cohort. Bars show Pearson correlation coefficients (r) between each axis gene (RAF–MEK–ERK or VEGF/VEGFR axis) and RAIR score; asterisks indicate BH-FDR significance (*FDR<0.05, **FDR<0.01, ***FDR<0.001).

(C) Drug-mechanism pathway support in GSE126698. Left: Pearson correlations between RAIR score and ssGSEA scores of pharmacology-relevant Hallmark pathways (KRAS signaling up, PI3K–AKT–mTOR signaling, and angiogenesis), with BH-FDR significance indicated by asterisks. Right: scatter plot of RAIR score versus KRAS signaling up ssGSEA score, with Pearson and Spearman correlations annotated and a fitted linear regression line.

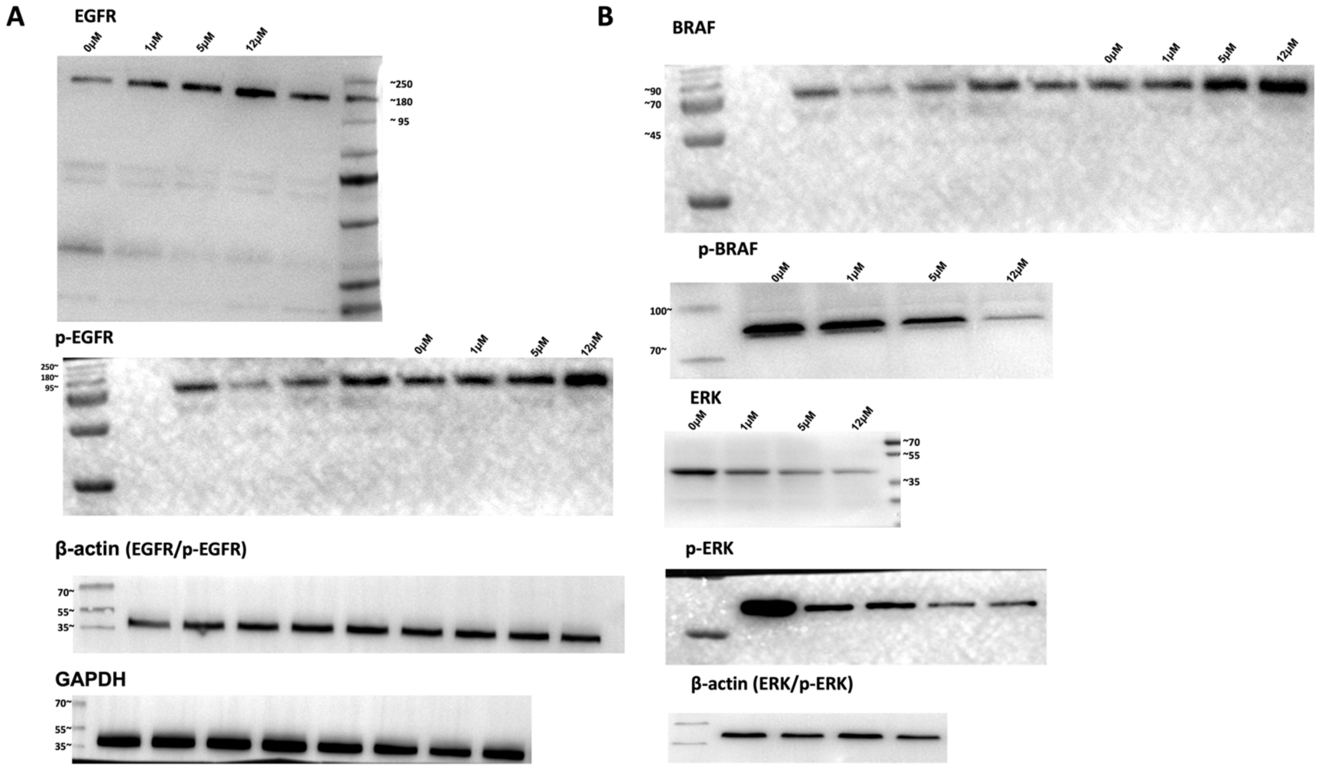

Figure S7. Uncropped immunoblots corresponding to Figure 11.

(A) Uncropped immunoblots showing total EGFR and phosphorylated EGFR (p-EGFR) in CAL-62 thyroid cancer cells treated with sorafenib at the indicated concentrations (0, 1, 5 and 12 μM). β-actin and GAPDH were used as loading controls for the corresponding EGFR/p-EGFR blots.

(B) Uncropped immunoblots showing total BRAF and phosphorylated BRAF (p-BRAF), and total ERK and phosphorylated ERK (p-ERK), in CAL-62 cells treated with the same sorafenib concentrations. β-actin was used as the loading control for the corresponding ERK/p-ERK blots.

Cells were treated for 6 h prior to protein extraction. Molecular weight markers (kDa) are indicated. These uncropped blots provide the original data supporting the cropped immunoblots and phospho/total-protein ratio quantification shown in Figure 11.

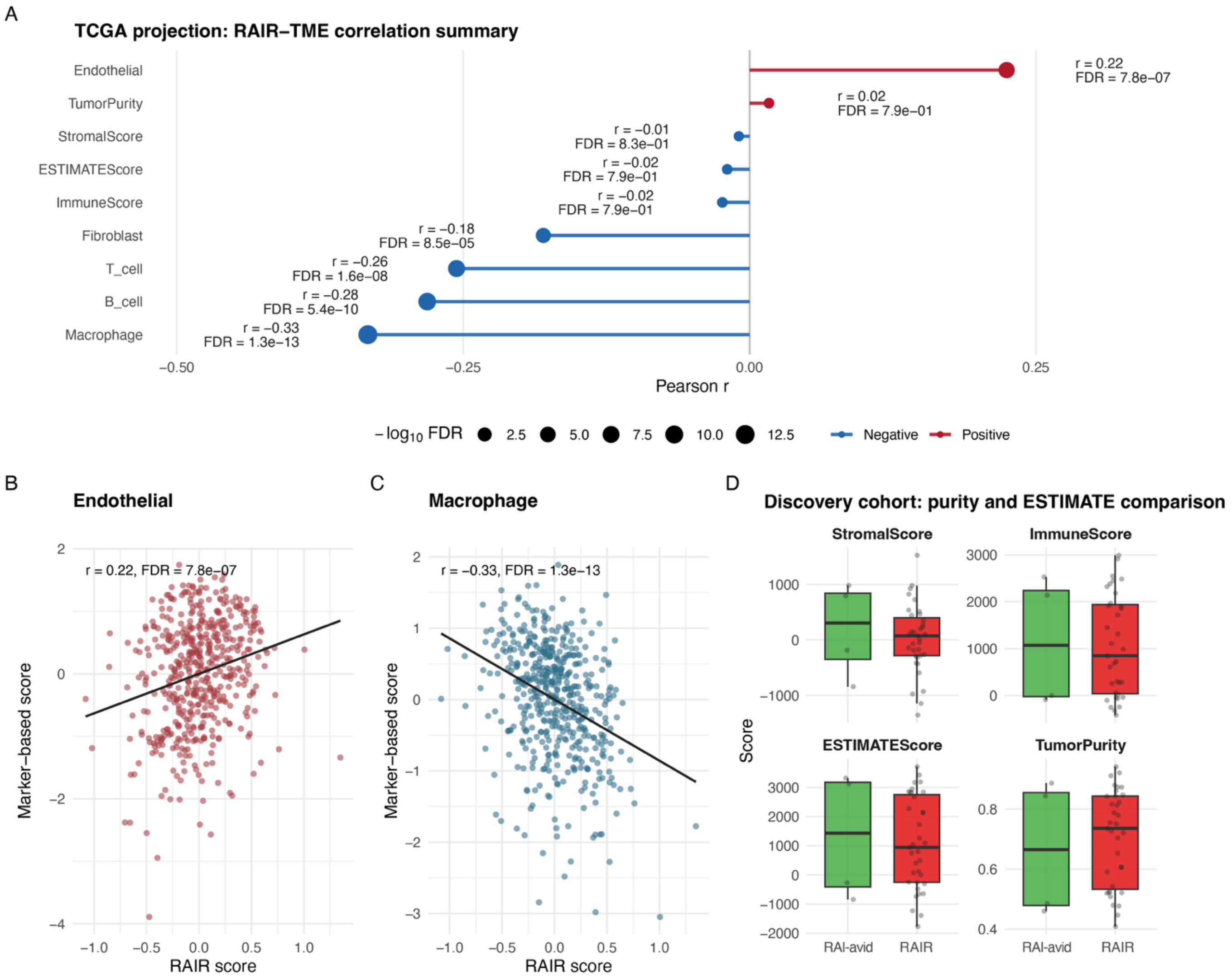

Figure S8. Tumour purity and tumour microenvironment-oriented projection analysis of the RAIR signature.

(A) Summary of Pearson correlations between the RAIR signature score and tumour microenvironment-related features in TCGA-THCA, including marker-based scores for macrophages, B cells, T cells, fibroblasts and endothelial cells, together with ESTIMATE-derived ImmuneScore, StromalScore, ESTIMATEScore and TumorPurity. Point size reflects −log10(FDR), and colours indicate the direction of correlation.

(B) Scatter plot showing the association between the RAIR signature score and the endothelial marker-based score in TCGA-THCA.

(C) Scatter plot showing the association between the RAIR signature score and the macrophage marker-based score in TCGA-THCA.

(D) Comparison of StromalScore, ImmuneScore, ESTIMATEScore and TumorPurity between RAI-avid and RAIR tumours in the discovery cohort GSE151179. Together, these analyses indicate that the RAIR signature is not primarily explained by overall bulk purity differences and instead captures a composite programme that includes selected microenvironment-linked signals.

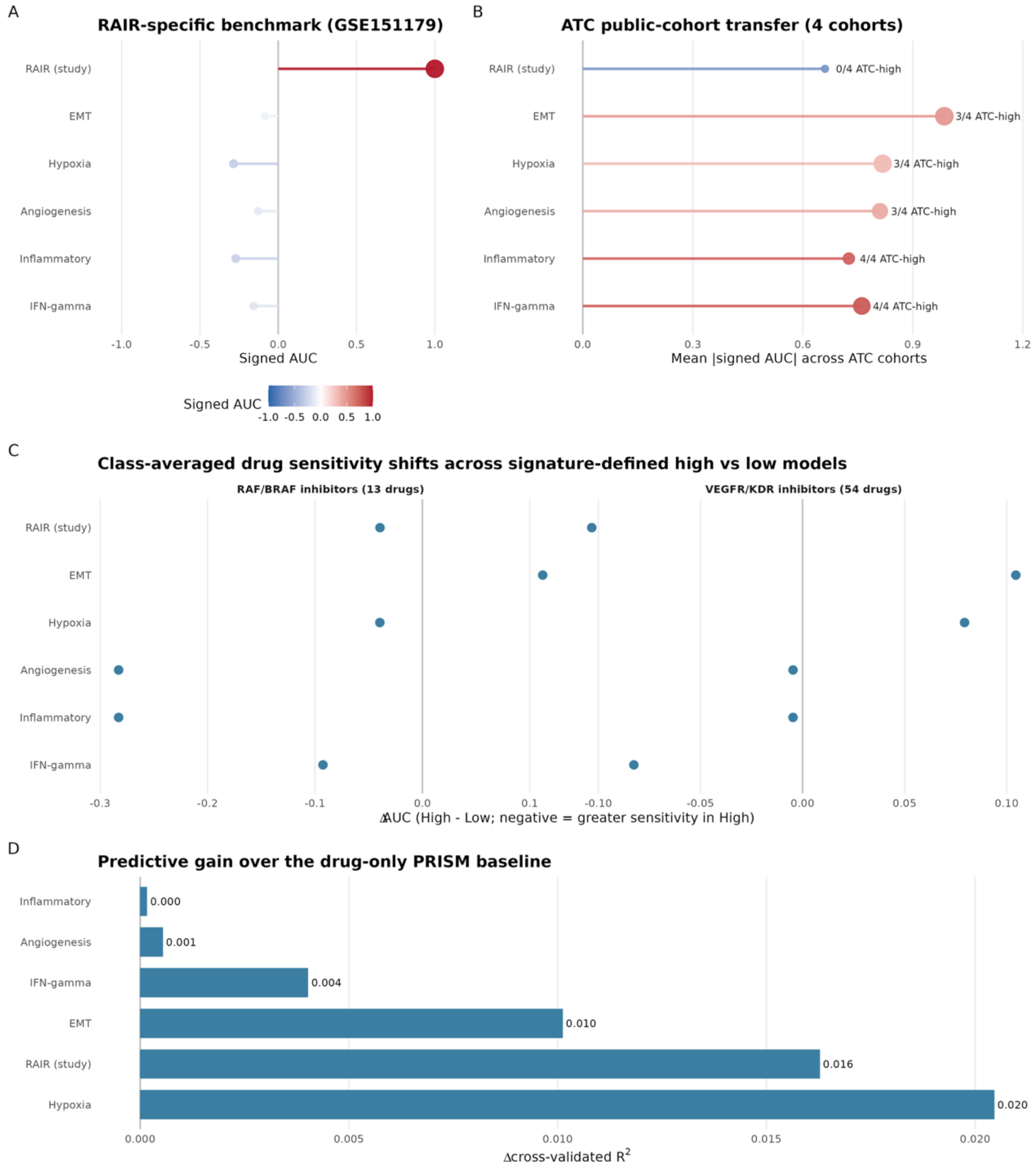

Figure S9. Benchmarking the RAIR signature against related transcriptional signatures across public cohorts, drug-sensitivity contrasts and PRISM prediction models.

(A) RAIR-specific benchmark in GSE151179. Signed AUC values were used to compare the ability of the study-derived RAIR signature and five related signatures (EMT, Hypoxia, Angiogenesis, Inflammatory and IFN-gamma) to distinguish radioiodine-refractory (RAIR) from RAI-avid tumours. Positive values indicate higher scores in RAIR tumours.

(B) Transfer of each signature to four independent ATC public cohorts. The x-axis shows the mean absolute signed AUC across cohorts, reflecting the overall magnitude of separation, and the annotation indicates the number of cohorts in which the signature was higher in ATC (k/4 ATC-high).

(C) Class-averaged drug-sensitivity shifts across signature-defined high versus low thyroid cancer cell-line models in DepMap/PRISM. Mean ΔAUC (High − Low) is shown for RAF/BRAF inhibitors and VEGFR/KDR inhibitors; negative values indicate lower PRISM AUC and therefore greater sensitivity in high-signature models.

(D) Predictive gain obtained by adding each signature to the drug-only PRISM baseline model. Bars show the increase in cross-validated R^2 after including the corresponding signature as an additional predictor. Together, these analyses indicate that the RAIR signature provides the strongest RAIR-specific discrimination and disease-focused value for VEGFR/KDR vulnerability prioritisation, while broader aggressive-state signatures such as hypoxia and EMT also contribute complementary predictive information.
